# Degradation of *YRA1* Pre-mRNA in the Cytoplasm Requires Translational Repression, Multiple Modular Intronic Elements, Edc3p, and Mex67p

**DOI:** 10.1371/journal.pbio.1000360

**Published:** 2010-04-27

**Authors:** Shuyun Dong, Allan Jacobson, Feng He

**Affiliations:** Department of Molecular Genetics and Microbiology, University of Massachusetts Medical School, Worcester, Massachusetts, United States of America; University of Wisconsin, United States of America

## Abstract

Intron-containing pre-mRNAs are normally retained and processed in the nucleus but are sometimes exported to the cytoplasm and degraded by the nonsense-mediated mRNA decay (NMD) pathway as a consequence of their inclusion of intronic in-frame termination codons. When shunted to the cytoplasm by autoregulated nuclear export, the intron-containing yeast *YRA1* pre-mRNA evades NMD and is targeted by a cytoplasmic decay pathway mediated by the decapping activator Edc3p. Here, we have elucidated this transcript-specific decay mechanism, showing that Edc3p-mediated *YRA1* pre-mRNA degradation occurs independently of translation and is controlled through five structurally distinct but functionally interdependent modular elements in the *YRA1* intron. Two of these elements target the pre-mRNA as an Edc3p substrate and the other three mediate transcript-specific translational repression. Translational repression of *YRA1* pre-mRNA also requires the heterodimeric Mex67p/Mtr2p general mRNA export receptor, but not Edc3p, and serves to enhance Edc3p substrate specificity by inhibiting the susceptibility of this pre-mRNA to NMD. Collectively, our data indicate that *YRA1* pre-mRNA degradation is a highly regulated process that proceeds through translational repression, substrate recognition by Edc3p, recruitment of the Dcp1p/Dcp2p decapping enzyme, and activation of decapping.

## Abstract 2

The yeast *YRA1* pre-mRNA contains multiple intronic elements that regulate transcript decay and translatability via the Edc3p decapping activator and the Mex67p/Mtr2p export receptor.

## Author Summary

Cellular mRNA levels are governed by competing rates of synthesis and decay. At the same time, mRNA decay pathways prevent the expression of defective mRNAs. The molecular mechanisms underlying the regulation of mRNA decay in eukaryotic cells are not well understood. We investigated a yeast transcript-specific decay pathway that targets the intron containing pre-mRNA for the mRNA export factor *Yra1p* when this pre-mRNA is shunted to the cytoplasm by autoregulated nuclear export. Our experiments demonstrate that the Edc3p decapping activator mediates *YRA1* pre-mRNA decay and that this process is independent of translation. Instead, it is controlled through five functionally interdependent modular elements contained in the *YRA1* intron. Whereas two of these elements confer Edc3p substrate specificity, the other three mediate translational repression of the *YRA1* pre-mRNA. Additionally, we found that translational repression of *YRA1* pre-mRNA requires Mex67p/Mtr2p, an mRNA export receptor, and enhances Edc3p substrate specificity by inhibiting the susceptibility of this pre-mRNA to nonsense-mediated mRNA decay. Our data highlight the intrinsic interconnections between different steps in gene expression and suggest that mRNA export factors in general may have important roles in controlling cytoplasmic mRNA translation and decay.

## Introduction

mRNA degradation controls the level of gene expression and ensures transcript quality control. In the yeast *Saccharomyces cerevisiae*, most wild-type mRNAs are degraded by the general 5′ to 3′ or 3′ to 5′ decay pathways [1]. Functionally impaired mRNAs are targeted for degradation by several translation-dependent mRNA surveillance mechanisms, including nonsense-mediated mRNA decay (NMD) for mRNAs containing premature termination codons [2], non-stop decay (NSD) for mRNAs lacking translation termination codons [3],[4], and no-go decay (NGD) for mRNAs stalled in translational elongation [5]. Transcript-specific decay pathways have also been identified in several experimental systems [6],[7]. In each of these pathways, degradation of a transcript is regulated by specific *cis*-acting elements and their respective *trans*-regulatory RNA-binding factors. For example, adenine/uridine-rich elements (AREs) have been found in the 3′-untranslated regions (3′-UTRs) of diverse eukaryotic mRNAs [8], and these elements by themselves, or through their interacting proteins, can accelerate transcript-specific decay by recruitment of the PARN and Ccr4p deadenylases [9],[10], the exosome [11]–[13], or the Dcp1p/Dcp2p decapping enzyme [9]. Our recent experiments, and those of Badis et al., have identified a yeast cytoplasmic, transcript-specific decay pathway, mediated by the decapping activator Edc3p, that principally targets only two transcripts, *RPS28B* mRNA and intron-containing *YRA1* pre-mRNA [14],[15].

Intron-containing pre-mRNAs are normally retained and processed in the nucleus [16],[17] but are sometimes exported to the cytoplasm where their inclusion of intronic in-frame termination codons targets these transcripts for degradation by the NMD pathway [18],[19]. However, the intron-containing *YRA1* pre-mRNA evades NMD and is degraded by the Edc3p-mediated decay pathway [15]. Importantly, this Edc3p-mediated *YRA1* pre-mRNA decay is dependent on the presence of the *YRA1* intron and appears to require the function of the general mRNA export factor Mex67p [15]. Here, we have dissected the intronic decay element and the role of Mex67p in Edc3p-mediated *YRA1* pre-mRNA decay. Our experiments delineated five structurally distinct but functionally interdependent *cis*-acting modules within the intron. Two modules dictate Edc3p substrate specificity and are designated as Edc3p responsive elements (EREs), whereas the other three modules, designated as translational repression elements (TREs), inhibit the translation of *YRA1* pre-mRNA. This translational repression requires Mex67p and Mtr2p, but not Edc3p, and prevents *YRA1* pre-mRNA from becoming a substrate for the NMD pathway.

## Results

### *YRA1* Pre-mRNA Is Translationally Repressed by a Mechanism Independent of Edc3p Activity

**Figure 1:**
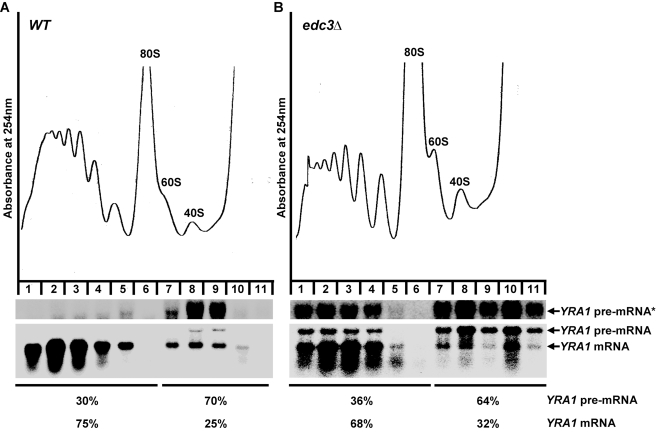
*YRA1* pre-mRNA is translationally repressed and Edc3p does not play a significant role in the repression mechanism. The polyribosomal association of *YRA1* pre-mRNA and mRNA in wild-type (A) and *edc3Δ* (B) cells was analyzed by sucrose gradient fractionation and Northern blotting. Upper panels: absorbance tracings at 254 nm; lower panels: Northern blots of individual gradient fractions. Blots were hybridized with a probe complementary to *YRA1* transcripts. The percentages of the *YRA1* pre-mRNA and mRNA in the mRNP and the polyribosomal fractions are indicated. Overexposed blots for enhanced *YRA1* pre-mRNA signals are indicated by an asterisk.

*YRA1* pre-mRNA contains multiple in-frame nonsense codons in its intron and, as such, could be considered to be a typical NMD substrate when present in the cytoplasm [18],[20]. Nevertheless, upon export from the nucleus, this pre-mRNA is degraded by the Edc3p-mediated decay pathway, not the NMD pathway [15]. Since NMD is dependent on translation, the NMD resistance of *YRA1* pre-mRNA could be attributable to translational repression of the transcript. Moreover, since *YRA1* pre-mRNA is still largely resistant to NMD in the absence of Edc3p [15], the hypothetical translational repression mechanism must not require Edc3p. To assess these possibilities, we analyzed the translation status of *YRA1* pre-mRNA in wild-type and *edc3Δ* strains utilizing sucrose gradient fractionation and northern blotting. In wild-type cells, the majority (70%) of the *YRA1* pre-mRNA population was present in the mRNP fractions, with only modest representation (30%) in the polyribosome fractions. In contrast, most (75%) of the *YRA1* mRNA was associated with the polyribosome fractions and only 25% was located in the mRNP fractions (Figure 1A). These results suggest that *YRA1* pre-mRNA is indeed translationally repressed in wild-type cells, whereas *YRA1* mRNA is not. Deletion of *EDC3* did not affect the overall polyribosome profile or significantly alter the distribution of *YRA1* pre-mRNA in the gradient. In *edc3Δ* cells, the majority (64%) of pre-mRNA transcripts was still present in the mRNP fractions. Likewise, the distribution of *YRA1* mRNA was essentially unaffected by deletion of *EDC3* (Figure 1B). These results indicate that, in spite of its cytoplasmic localization [15], *YRA1* pre-mRNA in wild-type or *edc3Δ* cells is translationally repressed, and Edc3p plays little role in establishing this repression.

### Edc3p-Mediated *YRA1* Pre-mRNA Degradation Does Not Require Translation

**Figure 2:**
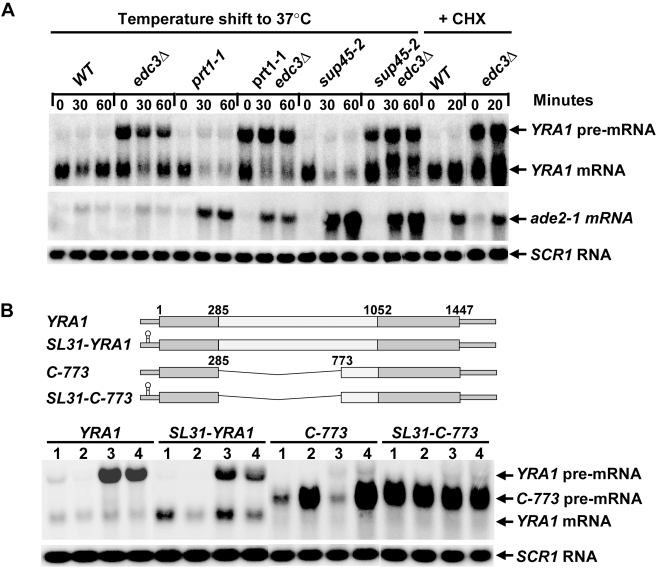
*Trans-* and *cis-*inhibition of translation have no effect on Edc3p-mediated *YRA1* pre-mRNA degradation. (A) Effects of *trans*-inhibition of translation on the steady-state levels of *YRA1* pre-mRNA and mRNA. Initiation was inhibited by inactivation of Prt1p, termination was inhibited by inactivation of Sup45p, and elongation was inhibited by treating cells with cycloheximide. At the indicated times post-inhibition, RNA was isolated from culture aliquots and subjected to Northern analysis. Blots were hybridized with probes complementary to the *YRA1*, *ADE2*, or *SCR1* transcripts, with the latter serving as a loading control. (B) The effects of *cis-*inhibition of translation initiation. A stem-loop structure was inserted into the 5′-UTRs of the *YRA1* gene or its *C-773* allele and the relative steady-state levels of the respective pre-mRNA and mRNA transcripts in wild-type (1), *upf1Δ* (2), *edc3Δ* (3), and *upf1Δedc3Δ* (4) cells were determined by Northern blotting as in (A). A schematic diagram of full-length *YRA1* pre-mRNA and the related transcripts derived from the *SL31-YRA1*, *C-773*, and *SL31-C-773* alleles is shown above the Northern blot. Smaller rectangles denote the 5′- and 3′-UTRs and larger rectangles denote the exons and the intron. The relative position of the 5′-UTR stem-loop structure is indicated, as are the nucleotides comprising the A of the initiator AUG (1), the 5′ (285) and 3′ (1052) boundaries of the intron, and the terminal nucleotide of the termination codon (1447).

The observed translational repression of *YRA1* pre-mRNA suggests that Edc3p-mediated *YRA1* pre-mRNA degradation occurs independently of ongoing protein synthesis. To test this notion, we carried out two sets of experiments. First, we examined the effects of *trans-*inhibition of translation initiation, elongation, or termination on *YRA1* pre-mRNA accumulation. In these experiments, initiation was inhibited by using the temperature-sensitive *prt1-1* allele to inactivate Prt1p, a component of the translation initiation factor eIF3 complex [21]; elongation was inhibited by treating cultures with the drug cycloheximide [22]; and termination was inhibited by using the temperature-sensitive *sup45-2* allele to inactivate Sup45p, the yeast eukaryotic release factor 1 (eRF1) [23]. We evaluated the effects of each of these three translation blocks in both *EDC3* and *edc3Δ* backgrounds and found that none of them affected the accumulation of *YRA1* pre-mRNA. While subjected to any of these three translation blocks, *EDC3* cells all accumulated low levels of *YRA1* pre-mRNA and *edc3Δ* cells all accumulated high levels of *YRA1* pre-mRNA (Figure 2A). As controls, we found that each of the three translation blocks caused 2- to 5-fold increases in the levels of nonsense-containing *ade2-1* mRNA (Figure 2A). These results show that inhibition of any of the three basic steps of translation has no effect on the degradation of *YRA1* pre-mRNA, i.e., even when general translation is inhibited *YRA1* pre-mRNA is still degraded in an Edc3p-dependent manner.

We also examined whether inclusion of a *cis*-acting inhibitor of translation had any effect on *YRA1* pre-mRNA decay. In this experiment, we inserted a stem-loop structure known to inhibit translation initiation [24] 31 nt upstream of the *YRA1* initiator AUG and analyzed the steady-state levels of the resulting *SL31-YRA1* pre-mRNA in wild-type, *upf1Δ*, *edc3Δ*, and *upf1Δedc3Δ* cells by northern blotting (the *UPF1* gene encodes a key component of the NMD pathway). We found that the *SL31-YRA1* pre-mRNA behaved like the wild-type pre-mRNA, i.e., it accumulated to low levels in wild-type or *upf1Δ* cells but increased 3- to 5-fold in *edc3Δ* or *upf1Δedc3Δ* cells (Figure 2B). As a control, we also inserted this stem-loop structure into the 5′-UTR of the *yra1 C-773* allele. This allele contains a large 5′ deletion of the *YRA1* intron and codes for a pre-mRNA that is degraded by NMD but not by Edc3p-mediated decay (Figure 2B). As expected, *SL31-C-773* pre-mRNA was stabilized more than 5-fold in cells with wild-type NMD function (Figure 2B). These data show that a *cis*-acting inhibitor of translation also fails to affect the decay of the *YRA1* pre-mRNA. Collectively, the experiments utilizing *cis*- or *trans*-inhibition of protein synthesis indicate that Edc3p-mediated *YRA1* pre-mRNA degradation does not require concomitant translation of the transcript and suggest that translational repression may be an important component of Edc3p-mediated *YRA1* pre-mRNA decay.

### Edc3p-Mediated *YRA1* Pre-mRNA Degradation Requires Multiple Elements of the *YRA1* Intron

We previously showed that Edc3p-mediated *YRA1* pre-mRNA degradation requires sequences within the *YRA1* intron [15]. To delineate the pertinent *cis*-regulatory elements, we generated a set of *YRA1* pre-mRNA transcripts harboring 3′ or 5′ deletions of the *YRA1* intron and analyzed the effects of each of these deletions on the steady-state levels of *YRA1* pre-mRNA in wild-type, *upf1Δ*, *edc3Δ*, and *upf1Δedc3Δ* cells. We included *upf1Δ* strains in these analyses because some intron deletions that inactivate Edc3p-mediated decay may simultaneously render a transcript sensitive to NMD, as each of these pre-mRNAs contains premature termination codons.

**Figure 3:**
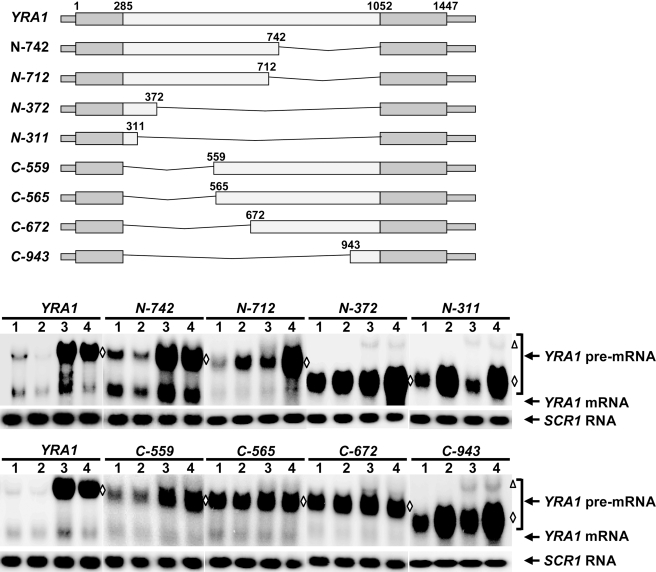
Effects of 5′ and 3′ deletions of the *YRA1* intron on Edc3p-mediated *YRA1* pre-mRNA decay. A set of *yra1* alleles containing 3′ or 5′ deletions of the *YRA1* intron was constructed and the steady-state levels of transcripts encoded by each of these alleles in wild-type (1), *upf1Δ* (2), *edc3Δ* (3), and *upf1Δedc3Δ* (4) cells were determined by Northern blotting. Blots were hybridized with probes complementary to the *YRA1* or *SCR1* transcripts, with the latter serving as a loading control. The positions of *YRA1* pre-mRNAs encoded by the endogenous and all the exogenous *YRA1* alleles are marked by a triangle and by diamonds, respectively. A schematic diagram of the *yra1* alleles analyzed is shown above the Northern blot, with the relative position of each deletion indicated. Pre-mRNAs encoded by each of the *YRA1* mutant alleles cannot be spliced to produce mRNAs, as the 5′ or 3′ splicing signals were deleted from these pre-mRNAs.

Deletions from the 3′ end of the intron yielded three distinct pre-mRNA decay phenotypes. First, deletions up to nt 742 (including alleles *N-742* in Figure 3, and *N-942*, *N-852*, *N-772*, and *N-752* in Figure S1) left the *YRA1* pre-mRNA decay phenotypes unchanged. Much like full-length wild-type *YRA1* pre-mRNA, the pre-mRNA transcripts encoded by these alleles showed increased accumulation (at least 5-fold) only in *edc3Δ* and *upf1Δedc3Δ* cells (Figure 3), indicating that these transcripts are still degraded exclusively by the Edc3p-mediated pathway. Second, further deletions from nt 742 to nt 372 (including alleles *N-712 and N-372* in Figure 3, and *N-542* and *N-400* in Figure S1) resulted in partial sensitivity to both NMD and Edc3p-mediated decay. The pre-mRNA transcripts encoded by these alleles showed modest increases (1.5- to 2-fold) in *upf1Δ* or *edc3Δ* strains but exhibited dramatic increases (more than 5-fold) in the *upf1Δedc3Δ* strain (Figures 3 and S1), indicating that these transcripts are degraded by either the Edc3p pathway or the NMD pathway. Since NMD requires translation, these results suggest that the region from nt 372 to 742 contains sequences that function in repressing *YRA1* pre-mRNA translation, thus inhibiting its degradation by NMD. Finally, further deletions from nt 372 (including alleles *N-311* in Figure 3 and *N-342* in Figure S1) resulted in sensitivity only to NMD but not to Edc3p-mediated decay. The pre-mRNA transcripts encoded by these alleles showed 4- to 5-fold increases in their respective levels only in *upf1Δ* and *upf1Δedc3Δ* cells (Figures 3 and S1), indicating that these transcripts are degraded exclusively by the NMD pathway. These results suggest that sequences 5′ of nt 372 of the *YRA1* intron are required for the Edc3p response. Since the smallest transcript capable of responding to Edc3p is that derived from construct *N-372*, which contains intron sequences from nt 286–372, this region must contain an ERE and we designate this segment as module A.

Deletions from the 5′ end of the intron up to nt 559 (including alleles *C-559* in Figure 3 and *C-395*, *C-543*, *C-548*, and *C-553* in Figure S2) did not affect the *YRA1* pre-mRNA decay phenotype. All of the pre-mRNA transcripts encoded by these alleles are degraded exclusively by the Edc3p pathway. Further 5′-end deletions, from nt 559 to nt 672 of the intron (including alleles *C-565* and *C-672* in Figure 3 and *C-625*, *C-637*, *C-648*, and *C-660* in Figure S2), eliminated the sensitivity to Edc3p-mediated decay. Interestingly, and in sharp contrast to the 3′ deletions described above, these deletions did not result in sensitivity to NMD. The pre-mRNA transcripts encoded by these alleles had 2- to 3-fold higher levels than full-length *YRA1* pre-mRNA in wild-type cells but exhibited similar levels in all four strains (Figures 3 and S2). These results indicate that the region between nt 559–672 of the *YRA1* intron is required for triggering Edc3p-mediated decay. This segment must also contain an ERE and we designate this region as module B.

Further 5′-end deletions, from nt 673 to 943 (including alleles *C-943* in Figure 3 and *C-678*, *C-683*, *C-713*, C-773, and *C-853* in Figure S2), resulted in sensitivity to Upf1p but not to Edc3p, indicating that these transcripts are primarily degraded by NMD. These results suggest that sequences downstream of nt 672 might function in repressing *YRA1* pre-mRNA translation and thus inhibit the transcript's degradation by NMD. Since the 3′ deletion analyses suggested that sequences upstream of nt 742 inhibit *YRA1* pre-mRNA degradation by NMD, the region from nt 672–742 is most likely involved in repressing *YRA1* pre-mRNA translation. Accordingly, we consider it to function as a TRE and designate this region as module C.

Taken together, these deletion analyses identified three intronic regions involved in the decay of *YRA1* pre-mRNA: modules A and B are required for the Edc3p response and module C appears to function in repressing *YRA1* pre-mRNA translation.

### *YRA1* Intron Modules Exhibit Synergistic and Partially Redundant Activities in Edc3p-Mediated *YRA1* Pre-mRNA Decay

**Figure 4:**
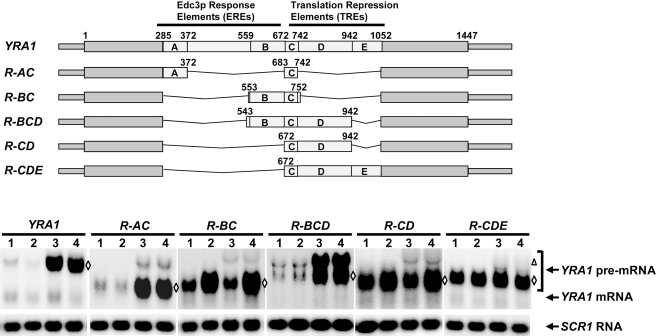
*YRA1* intron modules exhibit synergistic and partially redundant activities. A set of *yra1* alleles containing different combinations of *YRA1* intron modules was constructed and the steady-state levels of the transcripts encoded by each of these alleles in wild-type (1), *upf1Δ* (2), *edc3Δ* (3), and *upf1Δedc3Δ* (4) cells were determined by Northern blotting. Blots were hybridized with probes complementary to the *YRA1* or *SCR1* transcripts, with the latter serving as a loading control. The positions of *YRA1* pre-mRNAs encoded by the endogenous and all the exogenous *YRA1* alleles are marked by a triangle and by diamonds, respectively. A schematic diagram of the analyzed *yra1* alleles is shown above the Northern blot, with the relative positions and the implicated functions of modules A, B, C, D, and E indicated. Pre-mRNAs encoded by each of these recombinant *YRA1* alleles cannot be spliced to produce mRNAs, as they lack either the 5′ or the 3′ splicing signals, or both of these signals.

The results obtained with 3′ deletions of the intron implied that there is a functional dependency of ERE modules A and B on TRE module C, as a *YRA1* pre-mRNA containing ERE modules A and B but lacking TRE module C is partially susceptible to NMD (allele *N-712*, Figure 3). Interestingly, the two ERE modules do not share significant sequence homology and appear to have some functional difference. *YRA1* pre-mRNA containing module A alone is susceptible to both NMD and Edc3p-mediated decay (allele *N-372*, Figure 3). In contrast, *YRA1* pre-mRNA containing module B alone exhibits exclusive substrate specificity for NMD (allele *I-R4-NR2*, Figure S3). These observations raise the possibility that ERE modules A and B may have different requirements for TRE elements and perform at least partially redundant functions in Edc3p-mediated *YRA1* pre-mRNA decay. To assess this possibility, we generated *YRA1* pre-mRNAs containing different combinations of intron modules A, B, C, or additional intronic sequences, and analyzed the steady-state levels of the transcripts encoded by these alleles in wild-type, *upf1Δ*, *edc3Δ*, and *upf1Δedc3Δ* cells. Transcripts containing modules A and C exhibited the same specificity for the Edc3p-mediated pathway as did full-length *YRA1* pre-mRNA (allele *R-AC*, Figure 4). Transcripts containing modules B and C exhibited specificity for NMD but not for Edc3p-mediated decay (allele *R-BC*, Figure 4). Notably, transcripts containing modules B and C plus downstream sequences up to nt 942 exhibited specificity for Edc3p-mediated decay comparable to that manifested by full-length *YRA1* pre-mRNA (allele *R-BCD*, Figure 4). This result suggests that the segment downstream of module C, from nt 743 to nt 942, is also involved in Edc3p-mediated *YRA1* pre-mRNA degradation and we, therefore, designated this region as module D. Altogether, these data show that the ERE modules A and B indeed collaborate with different TRE modules and have at least partially redundant activities in Edc3p-mediated *YRA1* pre-mRNA decay.

Although the results described above implicated modules C and D in translational repression, transcripts containing modules C and D still exhibited specificity for NMD (allele *R-CD*, Figure 4). However, transcripts containing modules C and D plus downstream sequences up to nt 1052 are refractory to NMD (allele *R-CDE*, Figure 4). This result suggests that the segment downstream of module D, from nt 942–1052, also plays a role in repressing *YRA1* pre-mRNA translation. We designated this region as module E. As described below, modules C, D, and E can function together to repress *YRA1* pre-mRNA translation and they were, therefore, all designated as TREs.

Taken together, these experiments indicate that *YRA1* pre-mRNA degradation appears to involve five intronic sequence elements (Figure 4) and these five modules encompass two distinct functions. Modules A and B are required for the Edc3p response. Modules C, D, and E are not required for the Edc3p response *per se* but are most likely involved in repressing *YRA1* pre-mRNA translation since these modules together can inhibit the transcript's degradation by NMD. Importantly, a combination of modules A and C, or B, C, and D, is sufficient to trigger robust Edc3p-mediated decay. These results indicate that the respective ERE and TRE modules function synergistically in *YRA1* pre-mRNA decay.

### *YRA1* Intron Modules C, D, and E Mediate Translational Repression of *YRA1* Pre-mRNA

**Figure 5:**
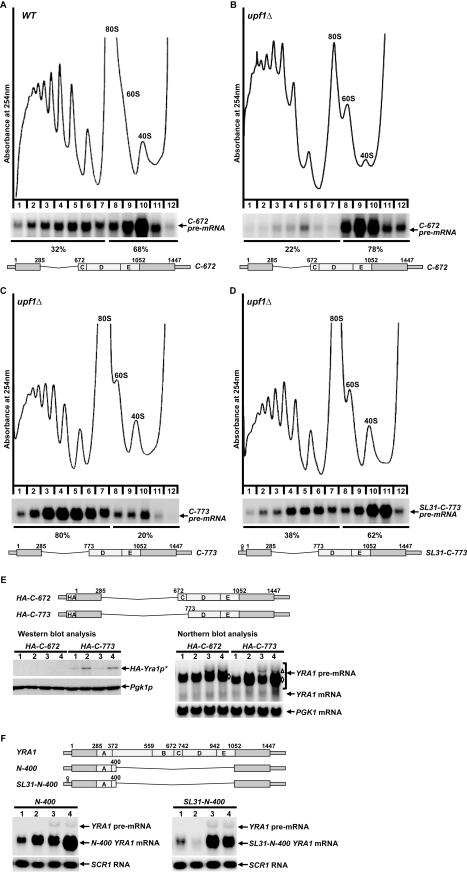
Intron modules C, D, and E mediate translational repression of *YRA1* pre-mRNA. (A–B) The polyribosomal association of the *YRA1* transcripts encoded by the *C-672* allele in wild-type cells (A) or *upf1Δ* cells (B) was analyzed by sucrose gradient fractionation and Northern blotting. Upper panels: absorbance tracings at 254 nm; middle panels: Northern blots of individual gradient fractions; lower panels: schematic diagrams of the *C-677* allele. Blots were hybridized with a probe complementary to *YRA1* transcripts. The percentages of the *C-672 YRA1* pre-mRNA present in the mRNP and polyribosomal fractions are indicated. (C–D) The polyribosomal association of the *YRA1* transcripts encoded by the *C-773* and *SL31-C-773* alleles in *upf1Δ* cells was analyzed by sucrose gradient fractionation and Northern blotting. Upper panels: absorbance tracings at 254 nm; middle panels: Northern blots of individual gradient fractions; lower panels: schematic diagrams of the *C-773 and SL31-C-773* alleles. Blots were hybridized with a probe complementary to *YRA1* transcripts. The percentages of the *C-773 or SL31-C-773 YRA1* pre-mRNAs present in the mRNP and polyribosomal fractions are indicated. (E) Analyses of steady-state RNA and protein expression from the *HA-C-672* and *HA-C-773* alleles in wild-type (1), *upf1Δ* (2), *edc3Δ* (3), and *upf1Δedc3Δ* (4) cells by Northern and Western blotting. Northern blots were hybridized with probes complementary to the *YRA1* or *PGK1* transcripts, with the latter serving as a loading control. The positions of the endogenous and exogenous *YRA1* pre-mRNAs are indicated by a triangle and by diamonds, respectively. Western blots of whole-cell extracts were probed with monoclonal antibodies against HA or Pgk1p, with the latter serving as a loading control. A schematic diagram of *HA-C-672* and *HA-C-773 YRA1* alleles is shown above the Northern and Western blots. The relative positions of the triple HA tag, the intron modules, and the intron deletions are indicated. Pre-mRNAs encoded by the *HA-C-672* and *HA-C-773 YRA1* alleles cannot be spliced to produce mRNAs, as they lack the 5′ splicing signals. (F) Analyses of the steady-state levels of *YRA1* pre-mRNAs encoded by the *N-400* and *SL31-N-400* alleles in wild-type (1), *upf1Δ* (2), *edc3Δ* (3), and *upf1Δedc3Δ* (4) cells by Northern blotting. Blots were hybridized with probes complementary to the *YRA1* or *SCR1* transcripts, with the latter serving as a loading control. The positions of the endogenous and exogenous *YRA1* pre-mRNAs are indicated. A schematic diagram of the wild-type, *N-400*, and *SL31-N-400 YRA1* alleles is shown above the Northern blot. The relative positions of the 5′-UTR stem-loop structure, the intron modules, and the intron deletions are indicated.

To further assess the role of modules C, D, and E in the translational repression of *YRA1* pre-mRNA, we examined the translation status of pre-mRNAs that contain or lack these modules in wild-type or *upf1Δ* cells. We analyzed two transcripts in this experiment. The first transcript, *C-672* pre-mRNA (Figure 5A and 5B), contains intact modules C, D, and E and is refractory to both Edc3p-mediated decay and NMD (Figure 3). The second transcript, *C-773* pre-mRNA (Figure 5C), is almost identical to *C-672* pre-mRNA except that it lacks module C and part of module D. This transcript is susceptible to NMD but not to Edc3p-mediated decay (Figure 2B). We analyzed the first transcript in wild-type and *upf1Δ* cells but the second transcript only in *upf1Δ* cells, because the second transcript is susceptible to NMD and has low abundance in wild-type cells. As shown in Figure 5A and 5B, the majority of the *C-672* transcript in both wild-type and *upf1Δ* cells was present in the mRNP fractions (68% in wild-type cells and 78% in *upf1Δ* cells), with only modest representation in the polyribosome fractions (32% in wild-type cells and 22% in *upf1Δ* cells). In contrast, the majority (80%) of the *C-773* transcript in *upf1Δ* cells was present in the polyribosome fractions, with only modest representation (20%) in the mRNP fractions (Figure 5C). As a control, we analyzed the translation status of *SL31-C-773* pre-mRNA in *upf1Δ* cells. *SL31-C773* pre-mRNA is identical to *C-773* pre-mRNA but harbors a stem-loop structure in its 5′-UTR that can inhibit translation initiation. In contrast to the *C-773* transcript (Figure 5C), the majority (62%) of the *SL31-C-773* transcript was present in the mRNP fractions, with only 38% in the polyribosome fractions (Figure 5D). This experiment shows that inclusion of a *cis*-inhibitory structure of translation initiation into the 5′-UTR of the *C-773* transcript causes a sizeable portion of that mRNA to shift from the polyribosome fractions to the mRNP fractions. These data show that the *C-672* transcript, which contains intact modules C, D, and E, is largely translationally repressed. In contrast, the *C-773* transcript, which contains only a part of module D and the entirety of module E, is actively engaged in translation.

To independently evaluate the above conclusion, we also constructed HA-tagged *C-672* and *C-773 yra1* alleles and analyzed their RNA and protein expression in wild-type, *upf1Δ*, *edc3Δ*, and *upf1Δedc3Δ* cells. The *HA-C-672* allele showed the same RNA expression pattern as the untagged *C-672* allele (compare allele *HA-C-672* in Figure 5E to allele *C-672* in Figure 3). The *YRA1* pre-mRNA encoded by the *HA-C-672* allele was insensitive to deletion of *UPF1* or *EDC3* and exhibited comparable high levels of expression in all four strains. Similarly, the *HA-C-773* allele showed the same RNA expression pattern as the untagged *C-773* allele (compare allele *HA-C773* in Figure 5E to allele *C-773* in Figure 2B). The *YRA1* pre-mRNA encoded by the *HA-C773* allele was sensitive to *UPF1* but not to *EDC3* and exhibited relatively low levels of expression in wild-type and *edc3Δ* cells but high levels in *upf1Δ* and *upf1Δedc3Δ* cells. However, despite the high levels of accumulation of the *HA-C-672* pre-mRNA in wild-type, *upf1Δ*, *edc3Δ*, and *upf1Δedc3Δ* cells, no protein expression was detected from this transcript (Figure 5E). In contrast, protein expression was detected from the *HA-C-773* pre-mRNA in all four strains (Figure 5E). Collectively, these experiments show that *YRA1* intron modules C, D, and E function in repressing *YRA1* pre-mRNA translation and suggest that this translational repression activity requires the combined actions of all three modules.

Our observation that intron modules C, D, and E function in repressing *YRA1* pre-mRNA translation further indicates that translational repression is a critical component of Edc3p-mediated *YRA1* pre-mRNA decay. However, the complexity of the *YRA1* intron elements and their functional interaction patterns raise the question of whether modules C, D, and E are only involved in translational repression or have additional regulatory functions (e.g., Edc3p substrate specificity). To address this issue, we tested whether *cis*-inhibition of translation initiation can suppress the defect caused by deletion of modules C, D, and E. In this experiment, we used the module A-containing *N-400* allele (Figure 5F). We inserted a stem-loop structure 31 nt upstream of the initiator AUG codon of the *N-400* allele to generate the *SL31-N-400* allele (Figure 5F) and analyzed the decay phenotypes of the transcripts encoded by this allele in wild-type, *upf1Δ*, *edc3Δ*, and *upf1Δedc3Δ* cells. Unlike the *N-400* pre-mRNA, which is a partial Edc3p substrate, the *SL31-N-400* pre-mRNA behaved like a *bona fide* Edc3p substrate (Figure 5F). These data show that *cis*-inhibition of translation initiation suppresses the defect caused by complete deletion of modules C, D, and E and fully restores the substrate status of an otherwise partial Edc3p substrate. These results indicate that *YRA1* intron modules C, D, and E function principally in repressing *YRA1* pre-mRNA translation and do not contribute to Edc3p substrate specificity directly.

### Mex67p Is a Component of the Cytoplasmic *YRA1* Pre-mRNP and Functions in Repressing *YRA1* Pre-mRNA Translation to Enhance Edc3p-Mediated Decay

**Figure 6:**
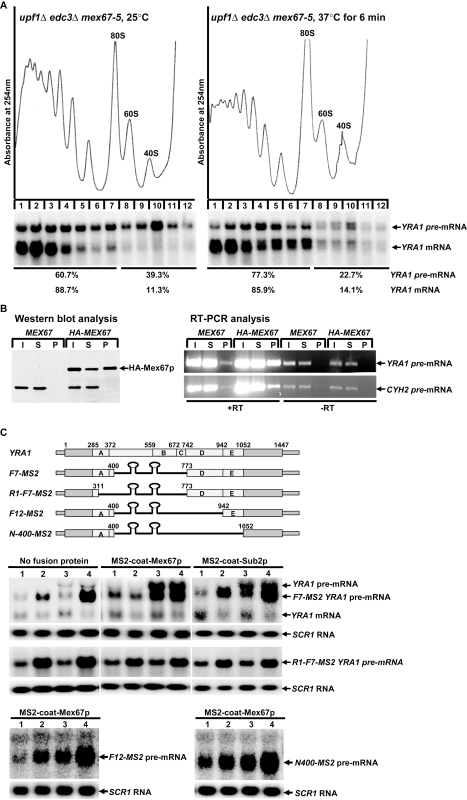
Mex67p is a component of the cytoplasmic *YRA1* pre-mRNP that functions in repressing *YRA1* pre-mRNA translation. (A) Analysis of the effect of inactivation of Mex67p on the translation of *YRA1* pre-mRNA. *upf1Δedc3Δmex67-5* cells were grown at 25°C and then shifted to 37°C for 6 min. The polyribosomal association of *YRA1* pre-mRNA and mRNA in these cells before or after the temperature shift was analyzed by sucrose gradient fractionation and Northern blotting. Upper panels: absorbance tracings at 254 nm; lower panels: Northern blots of individual gradient fractions. Blots were hybridized with a probe complementary to *YRA1* transcripts. The percentages of *YRA1* pre-mRNA and mRNA in the mRNP and the polyribosomal fractions are indicated. (B) Analysis of the association of Mex67p with *YRA1* pre-mRNA. Whole cell extracts from *upf1Δ edc3Δ* strains harboring either the *MEX67* or the HA-*MEX67* allele were incubated with anti-HA antibodies. Proteins and RNAs precipitated by the antibodies were analyzed by Western blotting (left panel) and RT-PCR (right panel). I, input; S, supernatant fraction; P, pellet fraction. HA-Mex67p and specific RT-PCR products for *YRA1* and *CYH2* pre-mRNAs were detected in the pellet fraction. RT, reverse transcriptase. (C) Analysis of the effect of tethering Mex67p on *YRA1* pre-mRNA decay. A DNA fragment containing two MS2-coat protein binding sites was inserted into the intronic region of the *F7*, *R1-F7*, *F12*, and *N-400* alleles of *YRA1*. The steady-state levels of the *YRA1* pre-mRNA transcripts encoded by the resulting *F7-MS2*, *R1-F7-MS2*, *F12-MS2*, *and N-400-MS2* alleles in wild-type (1), *upf1Δ* (2), *edc3Δ* (3), and *upf1Δedc3Δ* (4) cells that do or do not express the MS2-coat- Mex67p or Sub2p fusion proteins were determined by Northern blotting. Blots were hybridized with probes complementary to the *YRA1* or *SCR1* transcripts, with the latter serving as a loading control. The positions of the endogenous and exogenous *YRA1* pre-mRNAs and *YRA1* mRNA are indicated. A schematic diagram of the analyzed alleles is shown above the Northern blot, with the relative positions of the MS2-binding sites, the intron modules, and the intron deletions indicated.

We previously found that inactivation of Mex67p triggers rapid degradation of the *YRA1* pre-mRNA by NMD in *edc3Δ* cells [15]. Since NMD requires translation while Edc3p-mediated decay does not, we considered the possibility that Mex67p is a determinant of *YRA1* pre-mRNA translational repression. To assess the role of Mex67p in translational repression, we first analyzed the effect of inactivation of Mex67p on translation of *YRA1* pre-mRNA in *upf1Δedc3Δmex67-5* cells. As shown in Figure 6A, when cells were grown at the permissive temperature (25°C), a significant fraction of *YRA1* pre-mRNA is located in the mRNP fractions. However, when cells were shifted to the non-permissive temperature (37°C) for just 6 min, *YRA1* pre-mRNA originally located in the mRNP fractions moved to the polyribosome fractions. Importantly, this temperature shift did not cause the redistribution of the *YRA1* mRNA over the sucrose gradient. As a control, we also analyzed the translation status of *YRA1* pre-mRNA in *upf1Δedc3ΔMEX67* cells. We found that *YRA1* pre-mRNA in either the mRNP fractions or the polyribosome fractions remains unchanged during this 6-min temperature shift (Figure S4). These data show that inactivation of Mex67p mitigates the translational repression of *YRA1* pre-mRNA and suggest a direct role of Mex67p in translational control of *YRA1* pre-mRNA.

The apparent involvement of Mex67p in repressing *YRA1* pre-mRNA translation suggested that Mex67p binds to the *YRA1* pre-mRNA. To test this possibility, we constructed a yeast *upf1Δedc3Δ* strain harboring an HA-tagged *MEX67* allele and analyzed the association of Mex67p with *YRA1* pre-mRNA in this strain by co-immunoprecipitation and RT-PCR assays. As shown in Figure 6B, using anti-HA monoclonal antibodies, we were able to precipitate about 80% of HA-Mex67p fusion protein from whole-cell extracts and observed significant enrichment for *YRA1* pre-mRNA in the HA-Mex67p pellet. This result implies that Mex67p binds to *YRA1* pre-mRNA and is a component of the *YRA1* pre-mRNP.

To further address the role of Mex67p in translation repression of *YRA1* pre-mRNA, we tested whether tethering this protein can inhibit *YRA1* pre-mRNA degradation by NMD. In our initial experiment, we inserted two tandem MS2 coat protein binding sites into the intronic regions immediately upstream of module D sequences in the *F7* and *R1-F7* alleles and then analyzed the steady-state levels of the transcripts encoded by the resulting *F7-MS2* and *R1-F7-MS2* alleles in wild-type, *upf1Δ*, *edc3Δ*, and *upf1Δedc3Δ* cells which either express or do not express the MS2 coat protein/Mex67p fusion protein (MS2-coat-Mex67p). The *R1-F7* allele is almost identical to the *F7* allele except that it lacks a functional module A. The pre-mRNA transcript encoded by the *F7* allele is a substrate for NMD and Edc3p since this transcript is partially sensitive to deletion of either *UPF1* or *EDC3* (Figure S5). In contrast, the pre-mRNA transcript encoded by the *R1-F7* allele is a *bona fide* NMD substrate (Figure S5). In cells which did not express MS2-coat-Mex67p, the *F7-MS2* and *R1-F7-MS2* transcripts behaved the same as their counterpart transcripts lacking the MS2-binding sites (Figure 6C and Figure S5). The *F7-MS2* transcript was a substrate for both NMD and Edc3p and the *R1-F7-MS2* transcript was a substrate for NMD (Figure 6C). However, in cells which expressed MS2-coat-Mex67p, the *F7-MS2* transcript dramatically changed its decay phenotype. This transcript was no longer a substrate for NMD and behaved like a *bona fide* Edc3p substrate (Figure 6C). In contrast, the *R1-F7-MS2* transcript did not change its decay phenotype and maintained its status as an NMD substrate (Figure 6C). As controls, we also analyzed the decay phenotypes of *F7-MS2* and *R1-F7-MS2* pre-mRNAs in cells which expressed MS2 coat protein/Sub2p, Edc3p, or Crm1p fusion proteins. We found that the decay phenotypes of the *F7-MS2* and *R1-F7-MS2* transcripts remained unchanged in cells which expressed each of these fusion proteins (Figure 6C and unpublished data). These results show that tethering of Mex67p inhibits degradation of the transcript by NMD yet promotes its degradation by Edc3p-mediated decay. However, this NMD-inhibitory effect of tethering Mex67p is dependent on at least ERE module A.

To determine whether the NMD-inhibitory effect of tethering Mex67p requires additional *cis* elements besides the module A ERE, we analyzed two additional intron-containing *yra1* alleles, *F12-MS2* and *N-400-MS2*. These two alleles are identical to the *F7-MS2* allele except that *F12-MS2* lacks module D and *N-400-MS2* lacks both modules D and E. In contrast to the *F7-MS2* transcript, in cells which expressed MS2-coat-Mex67p, the transcripts encoded by the *F12-MS2* or *N-400-MS2* alleles remained as substrates for both NMD and Edc3p (Figure 6C). These results show that tethering Mex67p neither inhibited the degradation by NMD nor promoted the degradation by Edc3p for either of these transcripts. Taken together, our data show that tethering Mex67p to *YRA1* pre-mRNA can inhibit its translation and this inhibitory effect requires at least intron modules A and D.

We also find that, at least in *edc3Δ* cells, *YRA1* pre-mRNA localized to the polyribosome fractions co-sedimented with *YRA1* mRNA (Figure 1B), a surprising result that was also observed in additional experiments described in Figure 6A and Figure S4. Since *YRA1* pre-mRNA has a much shorter coding region than *YRA1* mRNA (285 nt versus 678 nt), these observations suggest that translationally repressed mRNPs have unusual compositions or conformations that may reflect blocks to more than one step in translation.

### Inactivation of Mtr2p Results in *YRA1* Pre-mRNA Degradation by NMD

**Figure 7:**
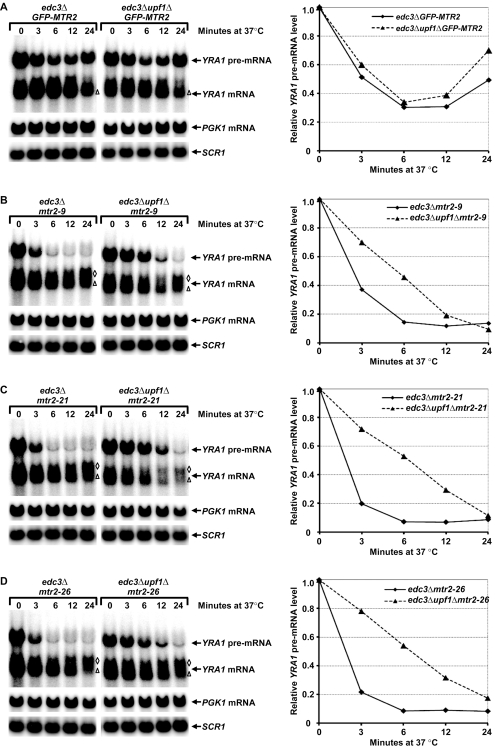
Inactivation of Mrt2p causes rapid degradation of *YRA1* pre-mRNA by NMD. *edc3Δ* and *edc3Δupf1Δ* cells harboring the fully functional *GFP-MTR2* (A) allele or the temperature-sensitive *mtr2–9* (B), *mtr2–21* (C), or *mtr2–26* (D) alleles were grown at the permissive temperature (25°C), then shifted to the restrictive temperature (37°C) for indicated times. Cells from each time point were collected and the levels of *YRA1* or *PGK1* transcripts were analyzed by Northern blotting. Blots were hybridized with probes complementary to the *YRA1*, *PGK1*, or *SCR1* transcripts, with the latter serving as a loading control. The positions of the normal *YRA1* mRNA species and the atypical longer *YRA1* mRNA species that accumulated in cells harboring the *mrt2–9*, *mtr2–21*, or *mtr2–26* alleles at late time points are indicated by a triangle and by diamonds, respectively. Graphs to the right of the figure depict *YRA1* pre-mRNA levels for each allele +/− Upf1p normalized to the corresponding “0” time point.

The results described in Figure 6 show that Mex67p plays a role in repressing *YRA1* pre-mRNA translation, inhibiting *YRA1* pre-mRNA degradation by NMD, and promoting the transcript's degradation by Edc3p. Since Mex67p and Mtr2p function as a complex in mRNA export [25], we sought to assess whether Mtr2p also plays a role in *YRA1* pre-mRNA decay. Accordingly, we constructed a set of *edc3Δ* and *upf1Δedc3Δ* strains harboring temperature-sensitive *mtr2* alleles and analyzed the effect of Mtr2p inactivation on the accumulation of *YRA1* pre-mRNA. We analyzed three different temperature-sensitive alleles (*mtr2–9*, *mtr2–21*, and *mtr2–26*) and included the fully functional GFP-tagged *MTR2* allele as a wild-type control. Previous studies had shown that the proteins encoded by these three temperature-sensitive alleles no longer interacted with Mex67p and at the restrictive temperature, cells harboring each of these alleles all manifested inhibition of nuclear mRNA export and mislocalization of Mex67p to cytoplasmic foci [25]. Our analyses indicated that, at the permissive temperature (25°C), *edc3Δ* cells harboring the *GFP-MTR2*, *mtr2–9*, *mtr2–21*, or *mtr2–26* alleles all accumulated comparably high levels of *YRA1* pre-mRNA (compare “0” time points, left panels in Figure 7A–D). However, when shifted to the restrictive temperature (37°C), *edc3Δ* cells harboring the *GFP-MTR2* allele behaved dramatically different from *edc3Δ* cells harboring the *mtr2–9*, *mtr2–21*, or *mtr2–26* alleles. During the 24-min time course, *edc3ΔGFP-MTR2* cells maintained relatively high levels of *YRA1* pre-mRNA at each time point (Figure 7A, left panel). In contrast, *edc3Δ* cells harboring the *mtr2–9*, *mtr2–21*, or *mtr2–26* alleles exhibited significant decreases in *YRA1* pre-mRNA level for each time point (compare the 3 6 12 and 24-min time points, Figure 7B–D left panels to that of Figure 7A). These decreases were transcript-specific since, during the 24-minute time course, *PGK1* mRNA levels remained unchanged in *edc3Δ* and *edc3Δupf1Δ* cells harboring the *GFP-MTR2*, *mtr2–9*, *mtr2–21*, or *mtr2–26* alleles (Figures 7A–D). Moreover, the dramatic decrease of *YRA1* pre-mRNA levels in *edc3Δ mtr2* cells was likely caused by rapid degradation of *YRA1* pre-mRNA by NMD, especially at the early time points (3 and 6 min), because deletion of *UPF1* mitigated these decreases (Figure 7B–D, right panels). Deletion of *UPF1* from *edc3Δmtr2–9*, *edc3Δmtr2–21*, and *edc3Δ mtr2–26* cells did not result in increased *YRA1* pre-mRNA accumulation at late time points (12 and 24 min, Figure 7B–D, right panels), suggesting that inactivation of Mtr2p may have also blocked *YRA1* pre-mRNA nuclear export, causing almost complete depletion of the cytoplasmic pool of *YRA1* pre-mRNA. Consistent with this interpretation, *mtr2–9*, *mtr2–21*, or *mtr2–26* cells in both *edc3Δ* and *edc3Δupf1Δ* backgrounds all accumulated a new, longer *YRA1* mRNA species at late time points of the temperature shift (12 and 24 min, Figure 7B–D). The accumulation of this new *YRA1* mRNA species is reminiscent of our previous observations in cells subject to Mex67p inactivation [15]. Taken together, the consequences of Mtr2p inactivation suggest that, similar to its role in general mRNA export, Mtr2p likely functions in a complex with Mex67p to repress *YRA1* pre-mRNA translation.

## Discussion

Edc3p-mediated *YRA1* pre-mRNA decay occurs in the cytoplasm [15]. In contrast to other cytoplasmic decay pathways, such as the NMD and general 5′ to 3′ decay pathways [1],[2], our data indicate that Edc3p-mediated *YRA1* pre-mRNA decay occurs independently of translation. This conclusion is supported by several observations, notably: (1) *YRA1* pre-mRNA is in a translationally repressed state in wild-type and *edc3Δ* cells (Figure 1); (2) *trans-*inhibition of general translation initiation, elongation, or termination has no significant effect on the steady-state levels of *YRA1* pre-mRNA in both *EDC3* and *edc3Δ* backgrounds (Figure 2A); and (3) inclusion of a *cis-*inhibitor of translation initiation in the 5′-UTR of *YRA1* pre-mRNA also has no effect on its decay (Figure 2B). Our finding that Edc3p-mediated *YRA1* pre-mRNA decay does not involve translation explains why this transcript is largely resistant to NMD despite the fact that it resembles a typical NMD substrate [20] and suggests that translational repression is a critical component of Edc3p-mediated *YRA1* pre-mRNA decay.

Our deletion analyses revealed five distinct modules important for Edc3p-mediated *YRA1* pre-mRNA decay. Modules A and B are required for triggering an Edc3p response and are thus *bona fide* EREs (Figures 3 and 4). Modules C, D, and E are required for inhibiting translation and function as TREs (Figures 4 and 5). Significantly, each of these modules except module A lacks an independent activity and appears to function synergistically with other modules essential for Edc3p-mediated *YRA1* pre-mRNA degradation (Figures 3, 4, and S3). Functional synergy was indeed manifested in several combinations of these modules. A combination of TRE modules C, D, and E can repress *YRA1* pre-mRNA translation (Figures 4 and 5) and two different combinations of ERE and TRE modules can trigger efficient Edc3p-mediated *YRA1* pre-mRNA decay (Figure 4). The underlying molecular mechanisms for these synergistic effects are currently not clear but may be indicative of cooperative binding to these intron modules by either different factors or different domains of the same factor. Interestingly, the two ERE modules (A and B) appear to lack synergistic activities and also have different functional requirements for TREs (Figures 4 and S6), suggesting that these two ERE modules perform at least partially redundant functions in Edc3p-mediated *YRA1* pre-mRNA decay (Figures 4 and S7).

Our observation that the *YRA1* intron contains two distinct functional elements indicates that the intron modules perform at least two essential functions in Edc3p-mediated *YRA1* pre-mRNA decay. The EREs appear to dictate Edc3p substrate specificity and most likely function to recruit Edc3p to the *YRA1* pre-mRNP. The TREs repress *YRA1* pre-mRNA translation and thus prevent degradation of the pre-mRNA by translation-dependent NMD and enhance its degradation by Edc3p. Since elimination of both EREs does not have any significant effects on TRE-mediated translational repression of *YRA1* pre-mRNA (Figure 5), and in contrast, the elimination of the three TREs causes partial loss of the ERE-mediated Edc3p response (Figure 3), these observations suggest that TRE-mediated translational repression functions upstream of ERE-mediated recruitment of Edc3p. When combined with the fact that Edc3p interacts with the Dcp1/Dcp2 decapping enzyme [26]–[33], these functional data indicate that Edc3p-mediated *YRA1* pre-mRNA degradation is most likely carried out through a series of ordered events including translational repression, recognition by Edc3p, recruitment of the decapping enzyme, and finally activation of decapping.

The complex *cis*-regulatory elements involved in Edc3p-mediated *YRA1* pre-mRNA decay are reminiscent of the localization elements identified in numerous mRNAs including *ASH1* mRNA in the yeast *Saccharomyces cerevisiae* [34], *nanos* mRNA in the fly *Drosophila melanogaster* [35],[36], and *Vg1* mRNA in the frog *Xenopus laevis* [37]. Like the *YRA1* intronic decay element, mRNA localization elements for each of these transcripts consist of multiple functionally distinct modules that can function independently but can also act synergistically to ensure proper mRNA localization. Importantly, translational repression is also an integral part of a localization element's function [34]. These observations suggest that mRNA decay may share common regulatory mechanisms with mRNA localization. Paradoxically, the *YRA1* intronic decay element bears no similarity to the *cis*-element regulating decay of the *RPS28B* mRNA, the only other known Edc3p substrate [14]. The *cis*-element of *RPS28B* mRNA is localized in its 3′-UTR and appears to form a single stem-loop structure that binds Rps28bp and recruits Edc3p [14]. These observations suggest that decay mechanisms may be significantly different even when transcripts are regulated by the same decay factor.

Several of our experiments demonstrate that the general mRNA exporter Mex67p is involved in repressing *YRA1* pre-mRNA translation. First, inactivation of Mex67p alleviates translational repression of *YRA1* pre-mRNA in *edc3Δupf1Δ* cells (Figure 6A). Second, Mex67p binds to *YRA1* pre-mRNA (Figure 6B). Third, tethering Mex67p to an NMD-susceptible partial Edc3p substrate inhibits the transcript's degradation by NMD and promotes its degradation by Edc3p-mediated decay (Figure 6C). Since the elimination of the TREs and inactivation of Mex67p have similar consequences on *YRA1* pre-mRNA decay, i.e., increased susceptibility to NMD and diminished susceptibility to Edc3p-mediated decay, one interesting possibility is that Mex67p binds directly to at least one of the TREs. In support of this idea, our experiments revealed that the NMD-inhibitory effect of tethering Mex67p also requires TRE module D besides ERE module A (Figure 6C). Interestingly, the human homolog of Mex67p, Tap, is also a sequence-specific RNA-binding protein that binds directly to constitutive transport elements (CTEs) of both viral and cellular intron-containing mRNAs [38]–[40]. Similar to its role in general mRNA export, Mex67p likely functions as a complex with Mtr2p in repressing *YRA1* pre-mRNA translation. Our finding that inactivation of Mex67p and Mtr2p both cause rapid degradation of *YRA1* pre-mRNA by NMD (Figure 7 and [15]) lends strong support for this conclusion.

A role for the Mex67p/Mtr2p general mRNA export factors in repressing *YRA1* pre-mRNA translation is intriguing and raises the possibility that these factors may also have a role in the translational control of additional mRNAs in yeast and in other eukaryotic cells. Consistent with this notion, the human homologues of Mex67p/Mtr2p (Tap/p15) have been shown to promote the translation of a CTE-containing mRNA [41] and the general yeast mRNA export factor, Gle1p, appears to interact with both translation initiation and termination factors and to regulate two distinct stages of translation [42]. Additionally, the general yeast mRNA export factor Dbp5p also exhibits genetic as well as physical interactions with translation termination factors [43]. These observations highlight the interconnections between different steps of the eukaryotic gene expression pathway and suggest that mRNA export factors may have a general as well as a specific role in controlling cytoplasmic mRNA translation and decay.

## Materials and Methods

### General Procedures

Most strains, protocols, and materials used in this study have been described previously [15]. Additional procedures used herein are summarized below.

### Yeast Strains

All strains used in this study are listed in Table S1. Strains containing deletions of *EDC3* or *UPF1* were constructed by gene replacement [44], using DNA fragments harboring the corresponding null alleles. Each genomic DNA deletion was confirmed by PCR analysis. Strains harboring the temperature-sensitive *prt1-1* or *sup45-2* alleles were constructed by the pop-in and pop-out technique [44]. Strains harboring the *GFP-MTR2* allele or the temperature-sensitive *mtr2–9*, *mtr2–21*, and *mtr2–26* alleles were constructed by plasmid shuffling [44].

### Plasmids

All plasmids used in this study are listed in Table S2. *YRA1* alleles harboring deletions of intron sequences, or containing insertions of a stem-loop structure, were generated by PCR and molecular cloning. *YRA1-MS2* constructs were prepared by annealing two oligonucleotides containing two tandem MS2-coat protein recognition sites and inserting the resulting DNA fragment into the *BamHI* and *EcoRI* sites in the intronic region of the *F7* and *R1-F7 YRA1* alleles. All *YRA1* alleles were confirmed by DNA sequencing. Plasmids expressing the MS2-coat protein fusions with Mex67p and Sub2p were generated by PCR and molecular cloning. In each case, a DNA fragment harboring the coding and 3′-UTR sequences of *MEX67* or *SUB2* was amplified using a pair of oligonucleotides that contain the *NheI* (5′ primer) and *SalI* (3′ primer) sites. The resulting DNA fragment was then inserted between the *NheI* and *SalI* sites of a plasmid that contains the *ADH1* promoter and the MS2 coding sequence. Each of these fusion proteins was expressed *in vivo* under the control of the *ADH1* promoter. HA-tagged *MEX67* allele was constructed by PCR and contains 410 bp from the promoter region of *MEX67*, 96 bp of triple HA, and 1,797 bp coding region and 260 bp 3′-UTR of *MEX67*. HA-tagged *YRA1 C-672* and *C-773* alleles were constructed by PCR. These two alleles are the same as their untagged alleles except that they contain a 101 bp *NcoI-NheI* fragment encoding a triple HA tag inserted at the initiation codon. The *HA-C-672* allele contains an in-frame stop codon six codons into the intron and *HA-C-773* contains an in-frame stop codon at the exon-intron junction.

### Oligonucleotides

The oligonucleotides used in this study were obtained from Operon, Inc., and are listed in Table S3.

### RNA Analysis

Cells were grown in either YEPD (Figures 2A and S8), or synthetic complete (SC) medium lacking tryptophan (Figures 2B, 3, 4, 5E, 5F, 6B, S1, S2, S3, S5, S6, and S7), leucine (Figures 7), or leucine and tryptophan (Figure 6C). For normal cell cultures, cells (15 ml) were grown at 30°C to an OD_600_ of 0.7 and harvested by centrifugation. For cultures involving cycloheximide treatment, cells (100 ml) were grown at 30°C to an OD_600_ of 0.7, harvested by centrifugation, and resuspended in 20 ml of the same medium. Cycloheximide was added to cell cultures at a final concentration of 100 µg/ml and 2 ml of cell cultures were harvested at different time points. For cultures involving temperature shifts, cells were grown at 25°C and treated as described previously [45]. In each case, cell pellets were frozen on dry ice and then stored at −80°C until RNA isolation. The procedures for RNA isolation and northern blotting were as previously described [45]. Transcript-specific signals were determined with a FUJI BAS-2500 analyzer.

### Protein Analysis

Preparation of whole-cell extracts and Western blotting procedures were as described previously [15]. Blots were probed with monoclonal antibodies against the HA epitope (12CA5, Roche) or against Pgk1p (22C5-D8, Molecular Probes), with the latter polypeptide serving as a loading control. Proteins were detected using ECL Western blotting detection reagents (GE Healthcare) and Kodak BioMax film.

### Polysome Analysis

Cells were grown at 30°C in either YEPD medium (Figure 1) or SC medium lacking tryptophan (Figures 5A–D, 6A, and S4) to an OD_600_ of 0.7. Cell extracts were prepared and fractionated on 7%*–*47% sucrose gradients as previously described [46].

### Analysis of RNAs Associated with Mex67p

Cells harboring an HA-tagged *MEX67* allele (100 ml) were grown at 30°C in SC medium lacking tryptophan to an OD_600_ of 0.7. Cells were collected by centrifugation, washed with ice-cold water, and resuspended in 1 ml of buffer B100 (10 mM Tris–HCl [pH 7.5], 100 mM KCl, 5 mM MgCl_2_, 1 mM dithiothreitol [DTT], 1 mM phenylmethylsulfonylfluoride, 10 mM Vanadyl ribonucleoside complex [VRC], 0.1% Nonidet P-40 [NP-40], 100 U/ml RNasin, and 1× protease inhibitor cocktail [Roche]). Cells were broken mechanically with glass beads and 50 µl extracts were incubated with 12 µl of a slurry of anti-HA agarose beads (Pierce) for 8 h at 4°C. The beads were washed four times with buffer B150 (same as B100 except containing150 mM KCl). RNA from the input extract and the supernatant and pellet fractions of the immunoprecipitation was isolated by phenol/chloroform extraction and ethanol precipitation. RNA from each of these samples was treated with DNase I and then analyzed by RT-PCR using a cDNA synthesis kit from Roche. Portions (1/25) of the input exact and the supernatant and pellet fractions were also analyzed by Western blotting to evaluate the efficiency of immuoprecipitating HA-Mex67p.

## Supporting Information

Figure S1 **Effects of 3′ deletions of the** ***YRA1*** **intron on Edc3p-mediated** ***YRA1*** **pre-mRNA decay.** A panel of *yra1* alleles containing deletions from the 3′-end of the *YRA1* intron was constructed and the steady-state levels of transcripts encoded by each of these alleles in wild-type (1), *upf1Δ* (2), *edc3Δ* (3), and *upf1Δedc3Δ* (4) cells were determined by Northern blotting. Blots were hybridized with probes complementary to the *YRA1* or *SCR1* transcripts, with the latter serving as a loading control. The positions of *YRA1* pre-mRNAs encoded by the endogenous and all the exogenous *YRA1* alleles are marked by a triangle and by diamonds, respectively. A schematic diagram of the analyzed *yra1* alleles is shown above the Northern blot, with the relative position of each deletion indicated. Pre-mRNAs encoded by each of the *YRA1* mutant alleles cannot be spliced to produce mRNAs, as the 3′ splicing signals were deleted from these pre-mRNAs. The transcripts are divided into three groups by broken lines based on their distinct decay phenotypes manifested in the Northern blots.
(0.98 MB TIF)

Figure S2 **Effects of 5′ deletions of the** ***YRA1*** **intron on Edc3p-mediated** ***YRA1*** **pre-mRNA decay.** A panel of *yra1* alleles containing deletions from the 5′-end of the *YRA1* intron was constructed and the steady-state levels of transcripts encoded by each of these alleles in wild-type (1), *upf1Δ* (2), *edc3Δ* (3), and *upf1Δedc3Δ* (4) cells were determined by Northern blotting. Blots were hybridized with probes complementary to the *YRA1* or *SCR1* transcripts, with the latter serving as a loading control. The positions of *YRA1* pre-mRNAs encoded by the endogenous and all the exogenous *YRA1* alleles are marked by a triangle and by diamonds, respectively. A schematic diagram of the analyzed *yra1* alleles is shown above the Northern blot, with the relative position of each deletion indicated. Pre-mRNAs encoded by each of the *YRA1* mutant alleles cannot be spliced to produce mRNAs, as the 5′ splicing signals were deleted from these pre-mRNAs. The transcripts are divided into three groups by broken lines based on their distinct decay phenotypes manifested in the Northern blots.
(2.44 MB TIF)

Figure S3 **Intronic modules B and C lack independent activity in Edc3p-mediated** ***YRA1*** **pre-mRNA decay.** A set of *yra1* alleles containing different internal fragments of module B and C regions of the *YRA1* intron was constructed and steady-state levels of the *YRA1* pre-mRNA encoded by each of these alleles in wild-type (1), *upf1Δ* (2), *edc3Δ* (3), and *upf1Δedc3Δ* (4) cells were determined by Northern blotting. Blots were hybridized with probes complementary to the *YRA1* transcript. The positions of *YRA1* pre-mRNAs encoded by the endogenous and all the exogenous *YRA1* alleles are marked by a triangle and by diamonds, respectively. A schematic diagram of the *yra1* alleles analyzed is shown above the Northern blot, with the starting and ending nt positions of each internal fragment indicated. Pre-mRNAs encoded by each of these *YRA1* mutant alleles cannot be spliced to produce mRNAs, as they lack both the 5′ and the 3′ splicing signals.
(0.50 MB TIF)

Figure S4 **A temperature shift does not alter the translation status of** ***YRA1*** **pre-mRNA in** ***upf1Δedc3ΔMEX67*** **cells.** *upf1Δedc3ΔMEX67* cells were grown at 25°C, shifted to 37°C for 6 min. The polyribosomal association of *YRA1* pre-mRNA and mRNA in these cells before (A) or after (B) the temperature shift was analyzed by sucrose gradient fractionation and Northern blotting. Upper panels: absorbance tracings at 254 nm; lower panels: Northern blots of individual gradient fractions. Blots were hybridized with a probe complementary to *YRA1* transcripts. The percentages of the *YRA1* pre-mRNA and mRNA in the mRNP and the polyribosomal fractions are indicated.
(1.29 MB TIF)

Figure S5 **Analysis of the decay phenotypes of the** ***YRA1*** **pre-mRNA transcripts encoded by the** ***yra1 F7*** **and** ***R1-F7*** **alleles.** *yra1* alleles harboring intronic deletions from nt 400 to 773 (F7) or from nt 311 to 773 (R1-F7) were constructed and the steady-state levels of the *YRA1* pre-mRNAs encoded by these alleles in wild-type (1), *upf1Δ* (2), *edc3Δ* (3), and *upf1Δedc3Δ* (4) cells were determined by Northern blotting. The blot was hybridized with probes complementary to the *YRA1* or *SCR1* transcripts, with the latter serving as a loading control. The positions of *YRA1* pre-mRNAs encoded by the endogenous and the exogenous alleles are marked by a triangle and by diamonds, respectively. A schematic diagram of the *F7* and *R1-F7* alleles analyzed is shown above the Northern blot, with the relative position of each deletion indicated. Pre-mRNAs encoded by both alleles can produce mRNAs because they still contain all the necessary splicing signals.
(0.35 MB TIF)

Figure S6 **ERE module A does not exhibit functional interaction with TRE modules D and E.** A set of *yra1* alleles containing different combinations of *YRA1* intron modules A, D, and E was constructed and the steady-state levels of transcripts encoded by each of these alleles in wild-type (1), *upf1Δ* (2), *edc3Δ* (3), and *upf1Δedc3Δ* (4) cells were determined by Northern blotting. Blots were hybridized with probes complementary to the *YRA1* transcript. The positions of *YRA1* pre-mRNAs encoded by the endogenous and all the exogenous *YRA1* alleles are marked by a triangle and by diamonds, respectively. A schematic diagram of the *yra1* alleles analyzed is shown above the Northern blot, with the relative positions of modules A, B, C, D, and E indicated. Pre-mRNAs encoded by each of these recombinant *YRA1* alleles can produce mRNAs as they still contain all the necessary splicing signals.
(0.47 MB TIF)

Figure S7 **Deletion of either module A or B does not affect Edc3p-mediated** ***YRA1*** **pre-mRNA decay.** A set of *yra1* alleles harboring deletions of either module A or B was constructed and the steady-state levels of the *YRA1* pre-mRNAs encoded by each of these alleles in wild-type (1), *upf1Δ* (2), *edc3Δ* (3), and *upf1Δedc3Δ* (4) cells were determined by Northern blotting. The blot was hybridized with a probe complementary to *YRA1* transcripts. The positions of *YRA1* pre-mRNAs encoded by the endogenous and all the exogenous *YRA1* alleles are marked by a triangle and by diamonds, respectively. A schematic diagram of the *yra1* alleles analyzed is shown above the Northern blot, with the relative position of each deletion indicated. Pre-mRNAs encoded by each of the *YRA1* mutant alleles can produce mRNAs because they still contain all the necessary splicing signals.
(0.50 MB TIF)

Figure S8 **Deletion of** ***UPF1*** **causes increased accumulation of** ***YRA1*** **pre-mRNA in** ***edc3Δ*** **cells.** Total RNA was isolated from wild-type (1), *upf1Δ* (2), *edc3Δ* (3), and *upf1Δedc3Δ* (4) and the steady-state levels of the *YRA1* pre-mRNA in these cells were determined by Northern blotting. The blot was hybridized with probes complementary to the *YRA1* or *SCR1* transcripts, with the latter serving as a loading control. The positions of *YRA1* pre-mRNA and mRNA are indicated.
(0.24 MB TIF)

Table S1 **Yeast strains used in this study.**
(0.09 MB PDF)

Table S2 **Plasmids used in this study.**
(0.10 MB PDF)

Table S3 **Oligonucleotides used in this study.**
(0.09 MB PDF)

## Abbreviations

AREs: adenine/uridine-rich elements
CTEs: constitutive transport elements
EREs: Edc3p responsive elements
eRF1: eukaryotic release factor 1
NGD: no-go decay
NMD: nonsense-mediated mRNA decay
NSD: non-stop decay
SC: synthetic complete
TREs: translational repression elements
UTR: untranslated region
