# Correction: Degradation of *YRA1* Pre-mRNA in the Cytoplasm Requires Translational Repression, Multiple Modular Intronic Elements, Edc3p, and Mex67p

**DOI:** 10.1371/journal.pbio.1002470

**Published:** 2016-05-12

**Authors:** Shuyun Dong, Allan Jacobson, Feng He

The authors would like to make the following clarifications regarding the manuscript figures, as well as provide a corrected version of Figure S1, provided here as S1 Fig.

- The authors would like to clarify that some of the figures in this article contain juxtaposed northern blot panels depicting distinct biological comparisons that have not been separated by vertical splice lines between the juxtaposed panels. Although splice lines were not indicated in those published figures, black lines or brackets were superimposed above panels to identify the distinct sets of gel lanes. The lack of splice lines does not affect any of the conclusions drawn from the experiments.
- The authors would also like to clarify some intentional duplications in the figures, which were done for purposes of comparison. Panels depicting the *YRA1*, *C-672*, *N-372*, *N-311*, *N-712*, and *C-943* northern blots in Figure 3 were included in the more extensive deletion series blots of Figures S1, S2, S3, S6 and S7 and the blot for the *C-672/R-CDE* synonymously named allele is included in both Figures 3 and 4 (referred to as C-672 in Figure 3 and as R-CDE in Figure 4).
- The authors acknowledge the intentional duplication of the Figure 5F *N400* panel in Figures S1 and S6, once again for purposes of comparison. The Figure S1 panel should have been associated with the corresponding *SCR1* blot shown in Figure 5F. Figure S1 has now been corrected accordingly and is provided here as S1 Fig. This correction does not affect the conclusions drawn in the study.

## Supporting Information

S1 Fig Effects of 3′ deletions of the *YRA1* intron on Edc3p-mediated *YRA1* pre-mRNA decay. A panel of *yra1* alleles containing deletions from the 3′-end of the *YRA1* intron was constructed and the steady-state levels of transcripts encoded by each of these alleles in wild-type (1), *upf1Δ* (2), *edc3Δ* (3), and *upf1Δedc3Δ* (4) cells were determined by northern blotting. Blots were hybridized with probes complementary to the *YRA1* or *SCR1* transcripts, with the latter serving as a loading control. The positions of *YRA1* pre-mRNAs encoded by the endogenous and all the exogenous *YRA1* alleles are marked by a triangle and by diamonds, respectively. A schematic diagram of the analyzed *yra1* alleles is shown above the northern blot, with the relative position of each deletion indicated. Pre-mRNAs encoded by each of the *YRA1* mutant alleles cannot be spliced to produce mRNAs, as the 3′ splicing signals were deleted from these pre-mRNAs. The transcripts are divided into three groups by broken lines based on their distinct decay phenotypes manifested in the northern blots.
(TIFF)
