# Supplementary figures and images for: Degradation of YRA1 Pre-mRNA in the Cytoplasm Requires Translational Repression, Multiple Modular Intronic Elements, Edc3p, and Mex67p
Source: PLoS Biol. 2010 Apr 27;8(4):e1000360. doi: 10.1371/journal.pbio.1000360 (PMC2864733; doi:10.1371/journal.pbio.1000360)

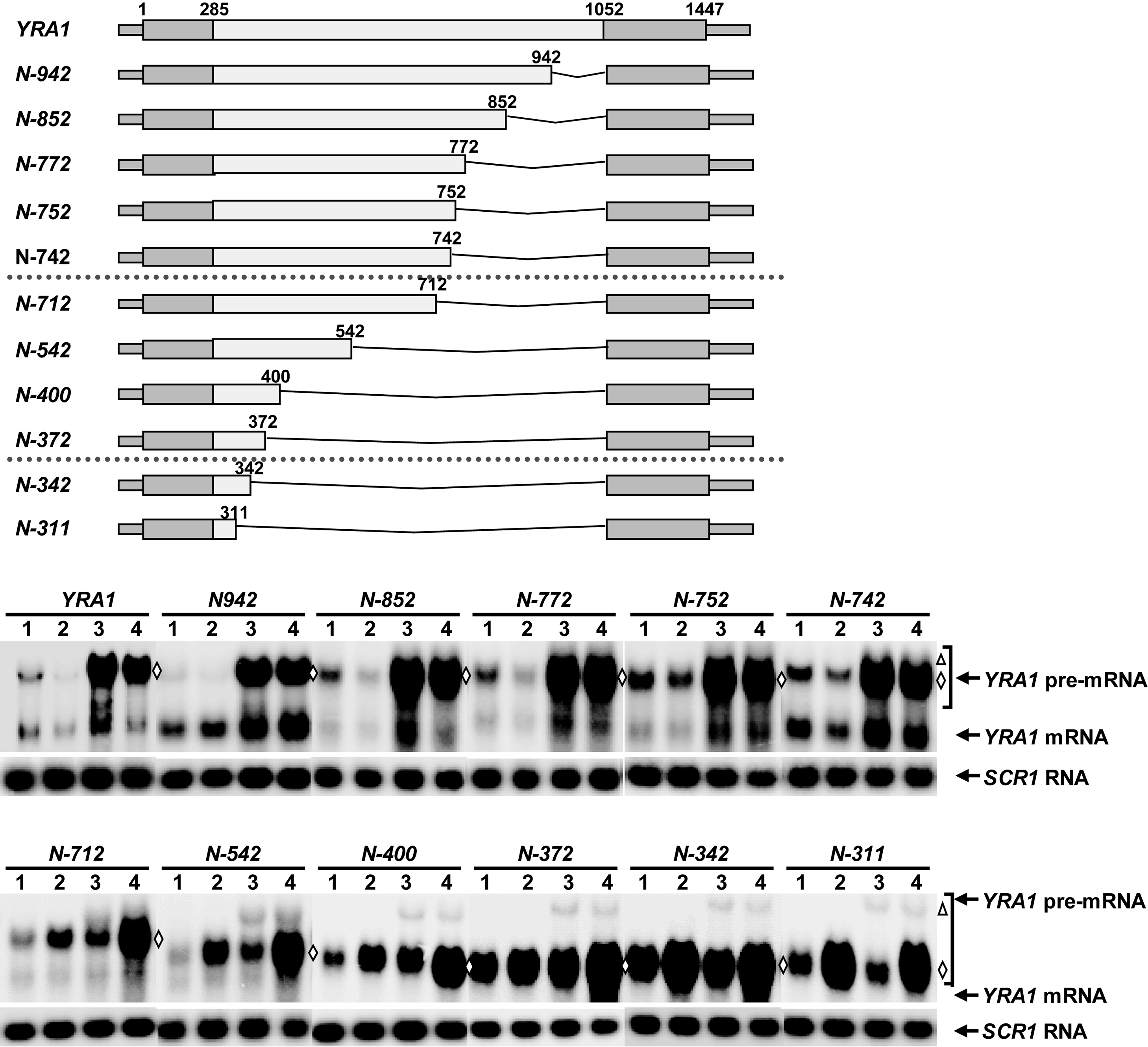

Supplement: Figure S1 — Effects of 3′ deletions of the YRA1 intron on Edc3p-mediated YRA1 pre-mRNA decay. A panel of yra1 alleles containing deletions from the 3′-end of the YRA1 intron was constructed and the steady-state levels of transcripts encoded by each of these alleles in wild-type (1), upf1Δ (2), edc3Δ (3), and upf1Δedc3Δ (4) cells were determined by Northern blotting. Blots were hybridized with probes complementary to the YRA1 or SCR1 transcripts, with the latter serving as a loading control. The positions of YRA1 pre-mRNAs encoded by the endogenous and all the exogenous YRA1 alleles are marked by a triangle and by diamonds, respectively. A schematic diagram of the analyzed yra1 alleles is shown above the Northern blot, with the relative position of each deletion indicated. Pre-mRNAs encoded by each of the YRA1 mutant alleles cannot be spliced to produce mRNAs, as the 3′ splicing signals were deleted from these pre-mRNAs. The transcripts are divided into three groups by broken lines based on their distinct decay phenotypes manifested in the Northern blots. (0.98 MB TIF) [file pbio.1000360.s001.tif]

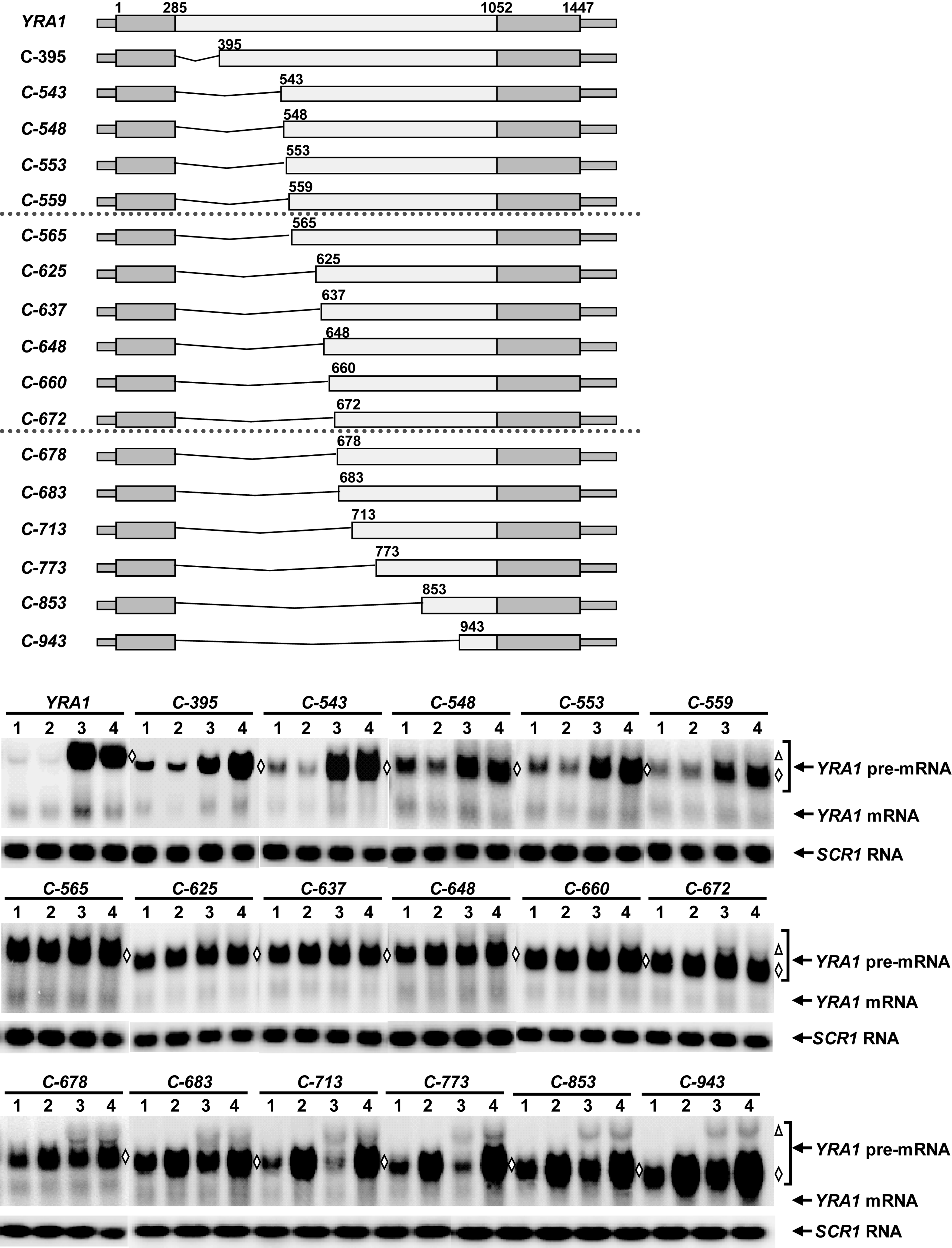

Supplement: Figure S2 — Effects of 5′ deletions of the YRA1 intron on Edc3p-mediated YRA1 pre-mRNA decay. A panel of yra1 alleles containing deletions from the 5′-end of the YRA1 intron was constructed and the steady-state levels of transcripts encoded by each of these alleles in wild-type (1), upf1Δ (2), edc3Δ (3), and upf1Δedc3Δ (4) cells were determined by Northern blotting. Blots were hybridized with probes complementary to the YRA1 or SCR1 transcripts, with the latter serving as a loading control. The positions of YRA1 pre-mRNAs encoded by the endogenous and all the exogenous YRA1 alleles are marked by a triangle and by diamonds, respectively. A schematic diagram of the analyzed yra1 alleles is shown above the Northern blot, with the relative position of each deletion indicated. Pre-mRNAs encoded by each of the YRA1 mutant alleles cannot be spliced to produce mRNAs, as the 5′ splicing signals were deleted from these pre-mRNAs. The transcripts are divided into three groups by broken lines based on their distinct decay phenotypes manifested in the Northern blots. (2.44 MB TIF) [file pbio.1000360.s002.tif]

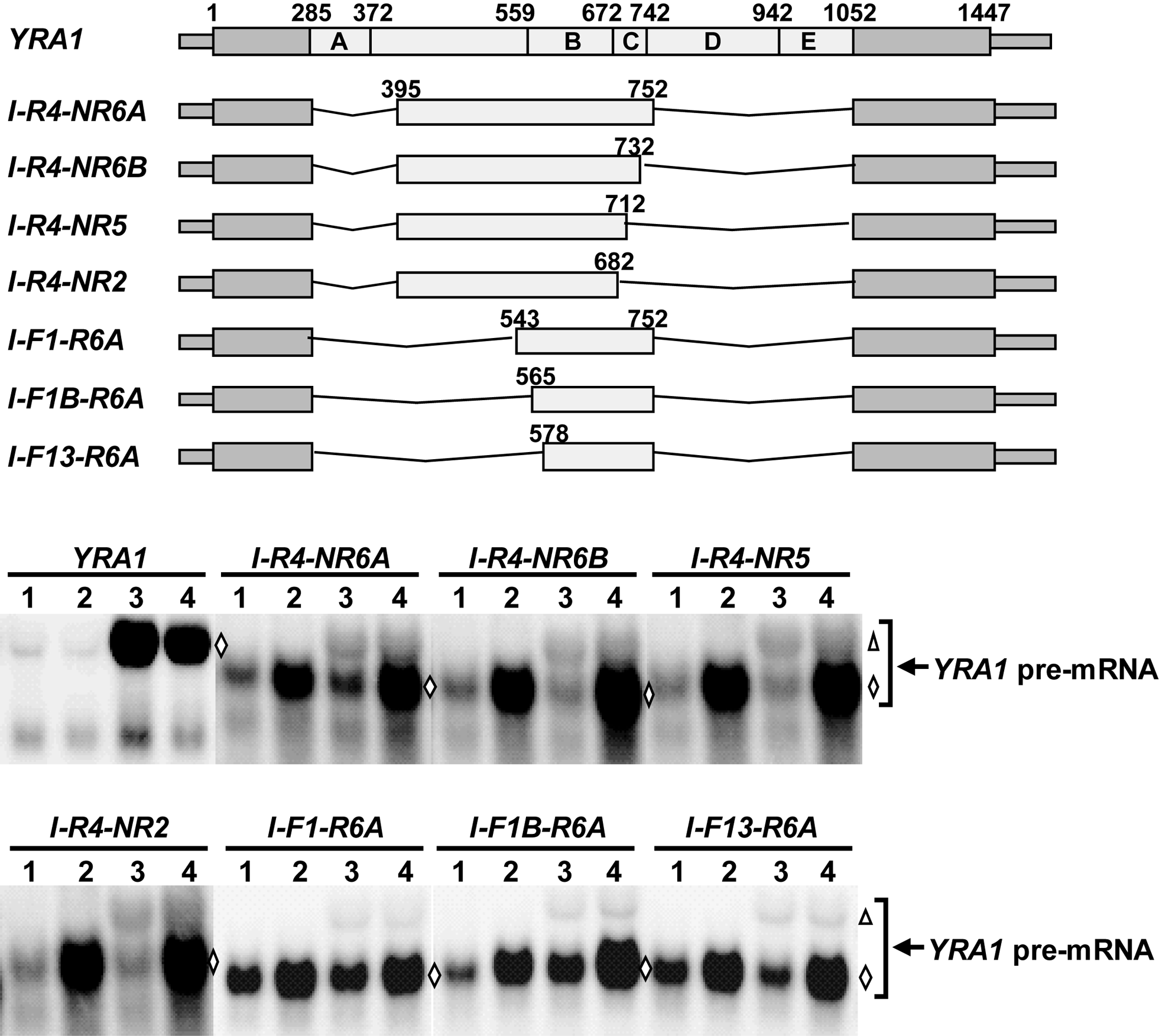

Supplement: Figure S3 — Intronic modules B and C lack independent activity in Edc3p-mediated YRA1 pre-mRNA decay. A set of yra1 alleles containing different internal fragments of module B and C regions of the YRA1 intron was constructed and steady-state levels of the YRA1 pre-mRNA encoded by each of these alleles in wild-type (1), upf1Δ (2), edc3Δ (3), and upf1Δedc3Δ (4) cells were determined by Northern blotting. Blots were hybridized with probes complementary to the YRA1 transcript. The positions of YRA1 pre-mRNAs encoded by the endogenous and all the exogenous YRA1 alleles are marked by a triangle and by diamonds, respectively. A schematic diagram of the yra1 alleles analyzed is shown above the Northern blot, with the starting and ending nt positions of each internal fragment indicated. Pre-mRNAs encoded by each of these YRA1 mutant alleles cannot be spliced to produce mRNAs, as they lack both the 5′ and the 3′ splicing signals. (0.50 MB TIF) [file pbio.1000360.s003.tif]

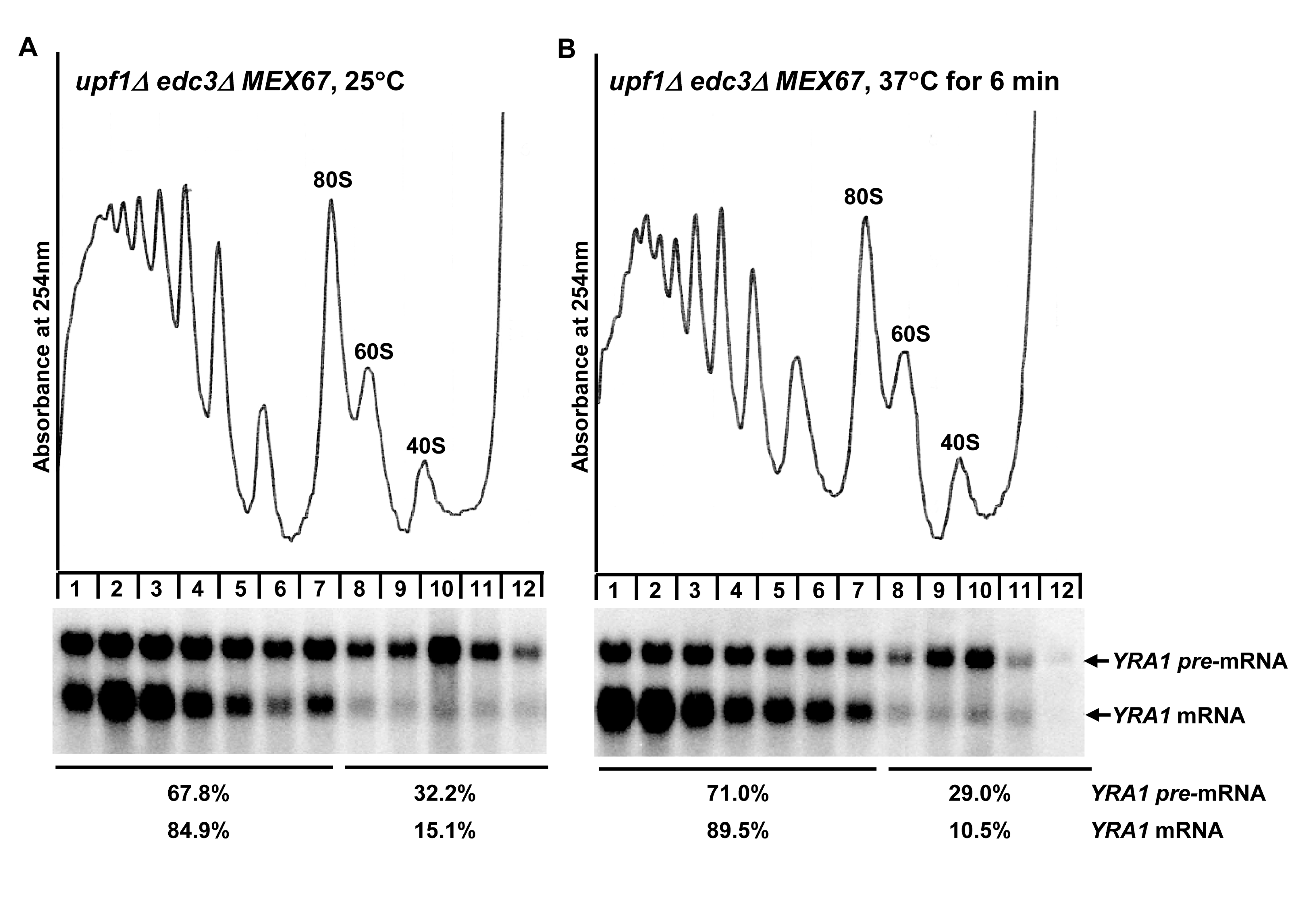

Supplement: Figure S4 — A temperature shift does not alter the translation status of YRA1 pre-mRNA in upf1Δedc3ΔMEX67 cells. upf1Δedc3ΔMEX67 cells were grown at 25°C, shifted to 37°C for 6 min. The polyribosomal association of YRA1 pre-mRNA and mRNA in these cells before (A) or after (B) the temperature shift was analyzed by sucrose gradient fractionation and Northern blotting. Upper panels: absorbance tracings at 254 nm; lower panels: Northern blots of individual gradient fractions. Blots were hybridized with a probe complementary to YRA1 transcripts. The percentages of the YRA1 pre-mRNA and mRNA in the mRNP and the polyribosomal fractions are indicated. (1.29 MB TIF) [file pbio.1000360.s004.tif]

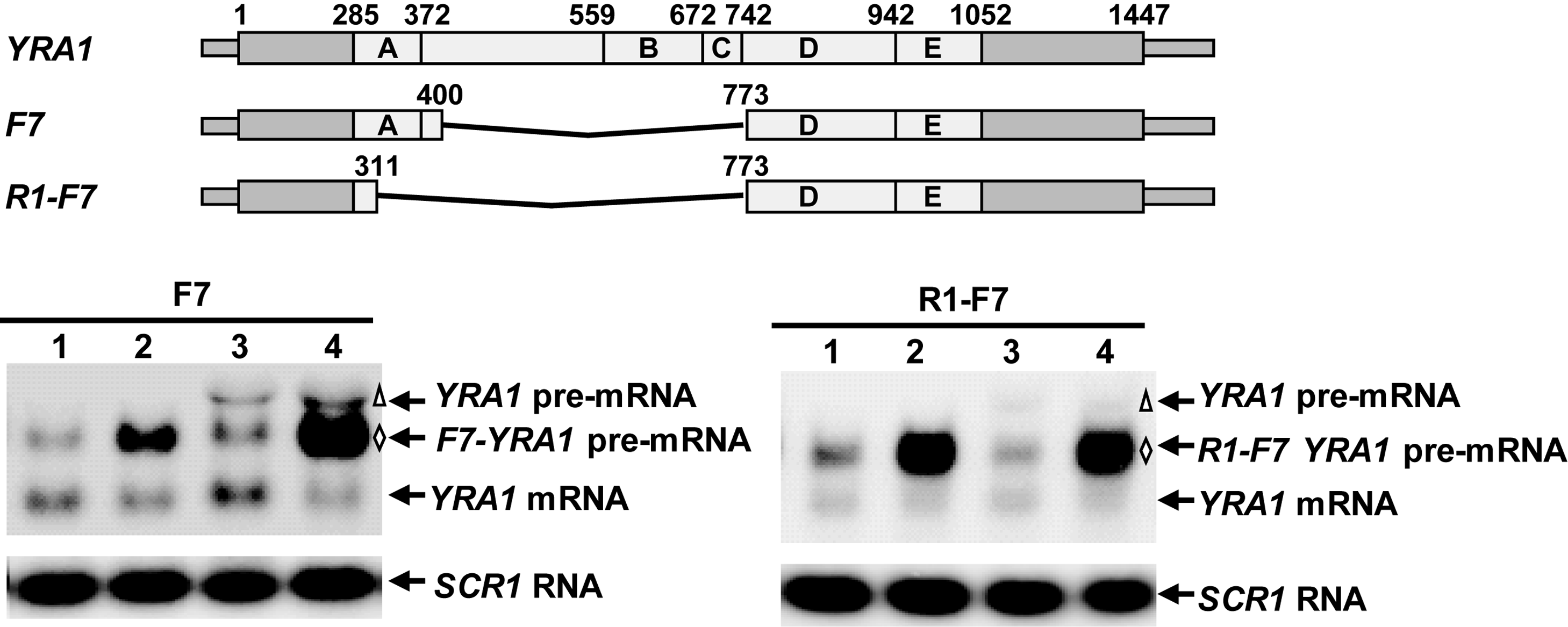

Supplement: Figure S5 — Analysis of the decay phenotypes of the YRA1 pre-mRNA transcripts encoded by the yra1 F7 and R1-F7 alleles. yra1 alleles harboring intronic deletions from nt 400 to 773 (F7) or from nt 311 to 773 (R1-F7) were constructed and the steady-state levels of the YRA1 pre-mRNAs encoded by these alleles in wild-type (1), upf1Δ (2), edc3Δ (3), and upf1Δedc3Δ (4) cells were determined by Northern blotting. The blot was hybridized with probes complementary to the YRA1 or SCR1 transcripts, with the latter serving as a loading control. The positions of YRA1 pre-mRNAs encoded by the endogenous and the exogenous alleles are marked by a triangle and by diamonds, respectively. A schematic diagram of the F7 and R1-F7 alleles analyzed is shown above the Northern blot, with the relative position of each deletion indicated. Pre-mRNAs encoded by both alleles can produce mRNAs because they still contain all the necessary splicing signals. (0.35 MB TIF) [file pbio.1000360.s005.tif]

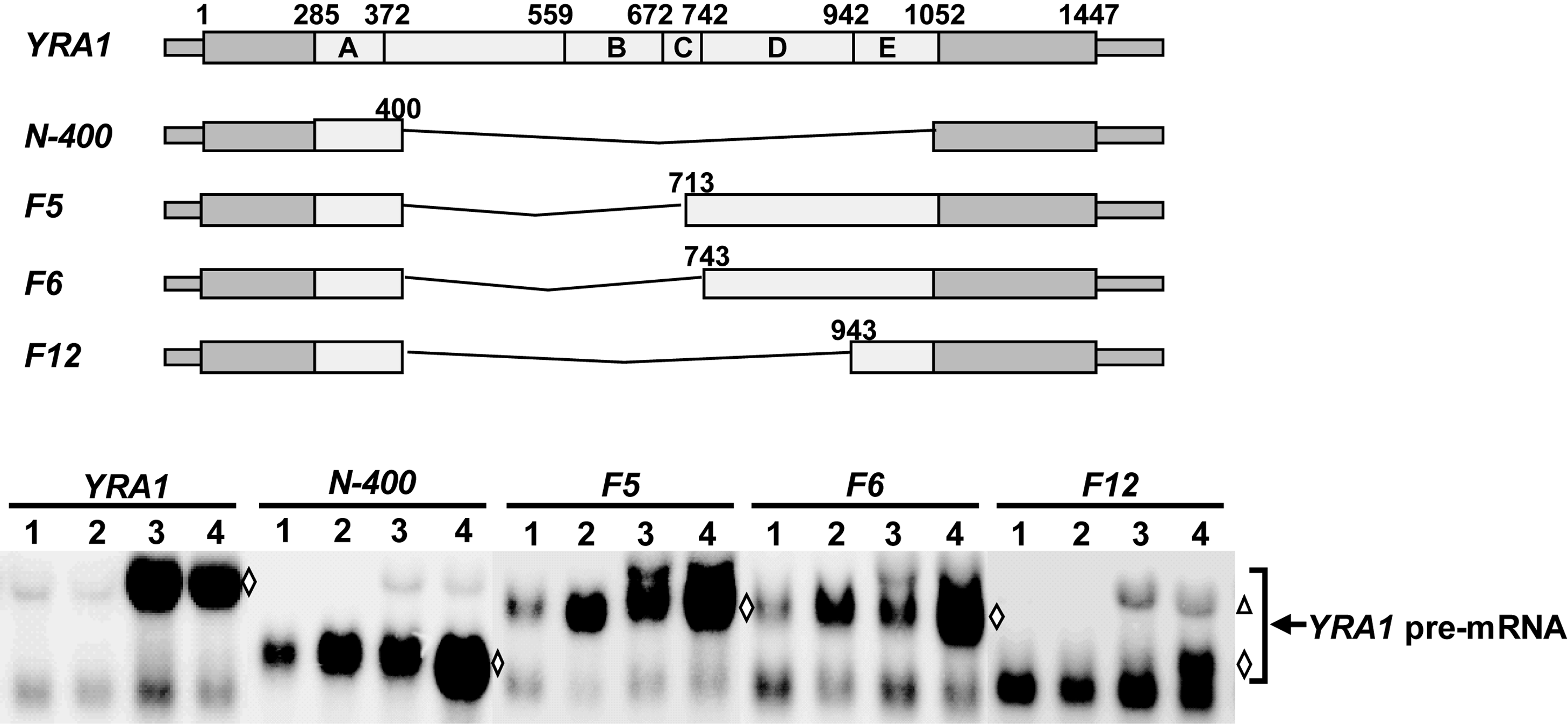

Supplement: Figure S6 — ERE module A does not exhibit functional interaction with TRE modules D and E. A set of yra1 alleles containing different combinations of YRA1 intron modules A, D, and E was constructed and the steady-state levels of transcripts encoded by each of these alleles in wild-type (1), upf1Δ (2), edc3Δ (3), and upf1Δedc3Δ (4) cells were determined by Northern blotting. Blots were hybridized with probes complementary to the YRA1 transcript. The positions of YRA1 pre-mRNAs encoded by the endogenous and all the exogenous YRA1 alleles are marked by a triangle and by diamonds, respectively. A schematic diagram of the yra1 alleles analyzed is shown above the Northern blot, with the relative positions of modules A, B, C, D, and E indicated. Pre-mRNAs encoded by each of these recombinant YRA1 alleles can produce mRNAs as they still contain all the necessary splicing signals. (0.47 MB TIF) [file pbio.1000360.s006.tif]

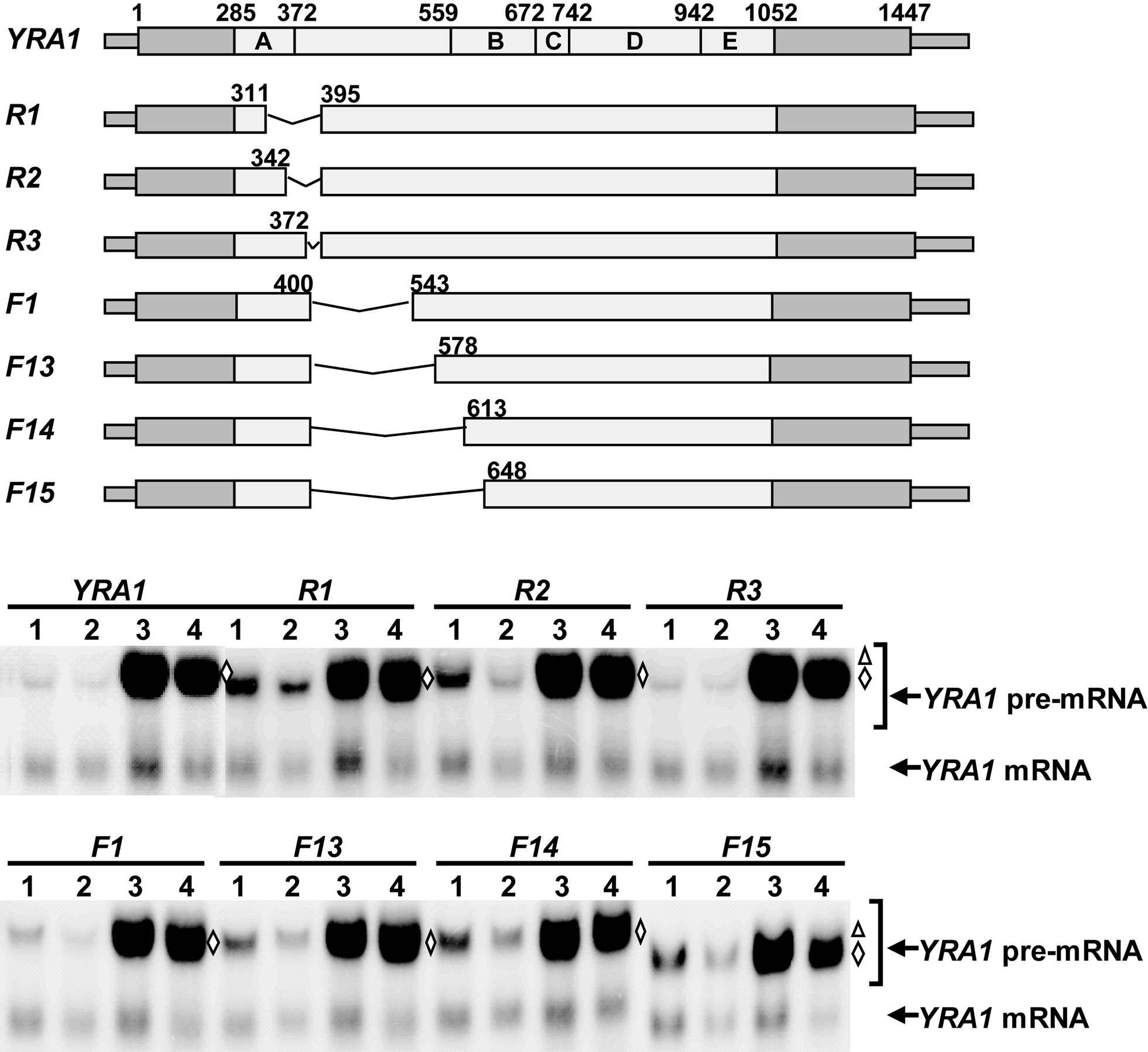

Supplement: Figure S7 — Deletion of either module A or B does not affect Edc3p-mediated YRA1 pre-mRNA decay. A set of yra1 alleles harboring deletions of either module A or B was constructed and the steady-state levels of the YRA1 pre-mRNAs encoded by each of these alleles in wild-type (1), upf1Δ (2), edc3Δ (3), and upf1Δedc3Δ (4) cells were determined by Northern blotting. The blot was hybridized with a probe complementary to YRA1 transcripts. The positions of YRA1 pre-mRNAs encoded by the endogenous and all the exogenous YRA1 alleles are marked by a triangle and by diamonds, respectively. A schematic diagram of the yra1 alleles analyzed is shown above the Northern blot, with the relative position of each deletion indicated. Pre-mRNAs encoded by each of the YRA1 mutant alleles can produce mRNAs because they still contain all the necessary splicing signals. (0.50 MB TIF) [file pbio.1000360.s007.tif]

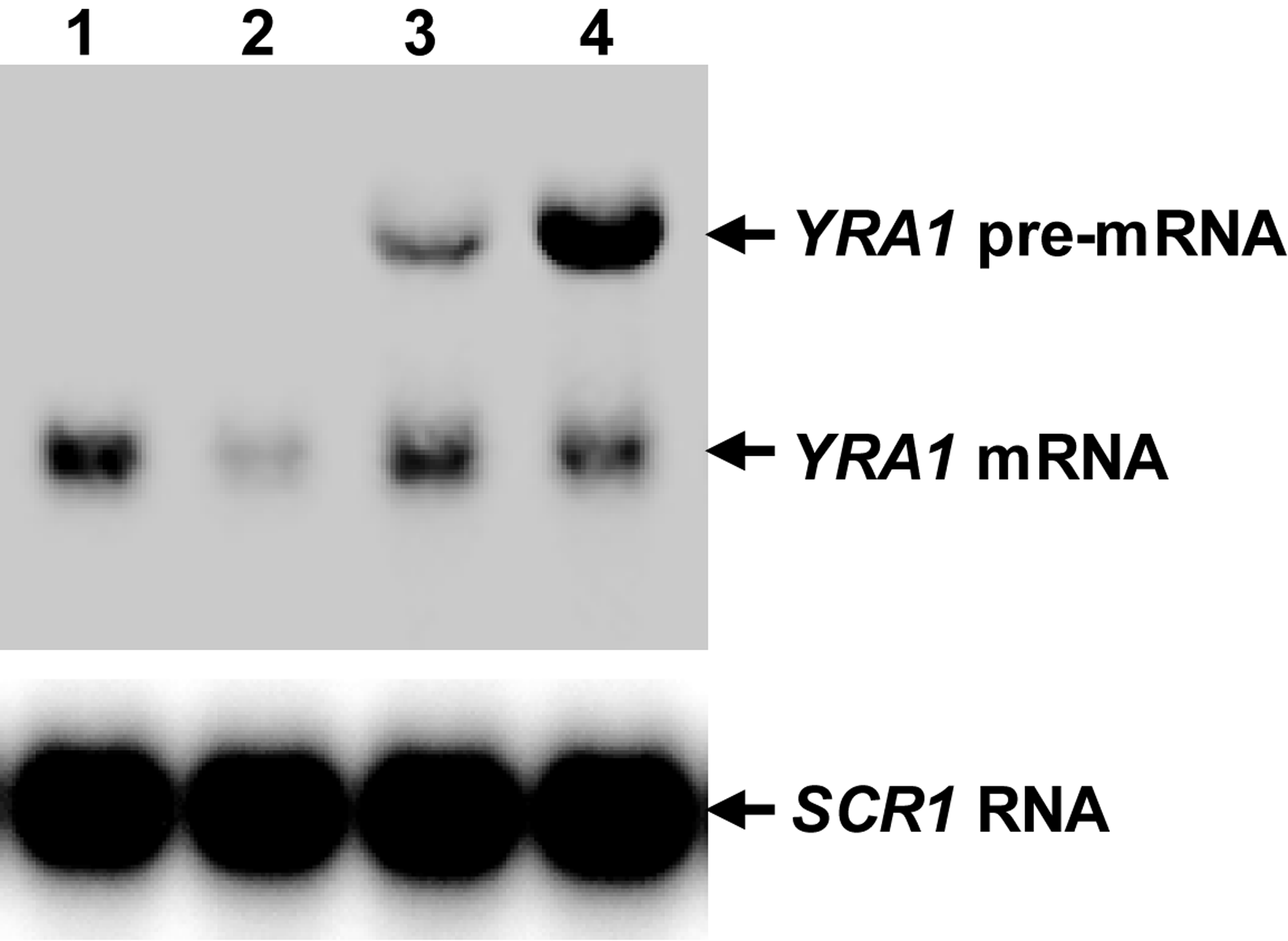

Supplement: Figure S8 — Deletion of UPF1 causes increased accumulation of YRA1 pre-mRNA in edc3Δ cells. Total RNA was isolated from wild-type (1), upf1Δ (2), edc3Δ (3), and upf1Δedc3Δ (4) and the steady-state levels of the YRA1 pre-mRNA in these cells were determined by Northern blotting. The blot was hybridized with probes complementary to the YRA1 or SCR1 transcripts, with the latter serving as a loading control. The positions of YRA1 pre-mRNA and mRNA are indicated. (0.24 MB TIF) [file pbio.1000360.s008.tif]
