# Supplementary material for: Degradation of YRA1 Pre-mRNA in the Cytoplasm Requires Translational Repression, Multiple Modular Intronic Elements, Edc3p, and Mex67p
Source: PLoS Biol. 2010 Apr 27;8(4):e1000360. doi: 10.1371/journal.pbio.1000360 (PMC2864733; doi:10.1371/journal.pbio.1000360)
Supplement: Table S1 — Yeast strains used in this study. (0.09 MB PDF) [file pbio.1000360.s009.pdf]

Name	Genotype
Y114	*MATa ade2-1 his3-11,15 leu2-3,112 trp1-1 ura3-1 can1-100 UPF1 NMD2 UPF3*
Y871	*MATa ade2-1 his3-11,15 leu2-3,112 trp1-1 ura3-1 can1-100 upf1::HIS3 NMD2 UPF3*
CFY25	*MATa ade2-1 his3-11,15 leu2-3,112 trp1-1 ura3-1 can1-100 UPF1 NMD2 UPF3 edc3::URA3*
SYY158	*MATa ade2-1 his3-11,15 leu2-3,112 trp1-1 ura3-1 can1-100 UPF1 NMD2 UPF3 edc3::URA3 upf1::HIS3*
CFY13	*MATa ade2-1 his3-11,15 leu2-3,112 trp1-1 ura3-1 can1-100 UPF1 NMD2 UPF3 prt1-1*
Y1218	*MATa ade2-1 his3-11,15 leu2-3,112 trp1-1 ura3-1 can1-100 UPF1 NMD2 UPF3 sup45-2*
SYY749	*MATa ade2 his3 leu2 trp1 ura3 mex67::HIS3 edc3::LEU2 upf1::URA3 [pRS314-mex67-5]*
SYY922	*MATa ade2 his3 leu2 trp1 ura3 MEX67 edc3::LEU2 upf1::URA3 [pRS314]*
SYY2330	*MATa ade2 his3 leu2 trp1 ura3 mex67::HIS3 edc3::LEU2 upf1::URA3 [pRS314-HA-MEX67]*
Y1029	*MATa ade2 his3 leu2 trp1 ura3 mtr2::HIS3 [pRS316-MTR2]*
SYY2360	*MATa ade2 his3 leu2 trp1 ura3 mtr2::HIS3 edc3::ADE2 [pRS316-MTR2]*
SYY2377	*MATa ade2 his3 leu2 trp1 ura3 mtr2::HIS3 edc3::ADE2 [pNOPGFP-MTR2]*
SYY2379	*MATa ade2 his3 leu2 trp1 ura3 mtr2::HIS3 edc3::ADE2 [pRS315-mtr2-9]*
SYY2381	*MATa ade2 his3 leu2 trp1 ura3 mtr2::HIS3 edc3::ADE2 [pRS315-mtr2-21]*
SYY2383	*MATa ade2 his3 leu2 trp1 ura3 mtr2::HIS3 edc3::ADE2 [pRS315-mtr2-26]*
SYY2363	*MATa ade2 his3 leu2 trp1 ura3 mtr2::HIS3 edc3::ADE2 upf1::KanMX6 [pRS316-MTR2]*
SYY2369	*MATa ade2 his3 leu2 trp1 ura3 mtr2::HIS3 edc3::ADE2 upf1::KanMX6 [pNOPGFP-MTR2]*
SYY2371	*MATa ade2 his3 leu2 trp1 ura3 mtr2::HIS3 edc3::ADE2 upf1::KanMX6 [pRS315-mtr2-9]*
SYY2373	*MATa ade2 his3 leu2 trp1 ura3 mtr2::HIS3 edc3::ADE2 upf1::KanMX6 [pRS315-mtr2-21]*
SYY2375	*MATa ade2 his3 leu2 trp1 ura3 mtr2::HIS3 edc3::ADE2 upf1::KanMX6 [pRS315-mtr2-26]*
