# Supplementary material for: Degradation of YRA1 Pre-mRNA in the Cytoplasm Requires Translational Repression, Multiple Modular Intronic Elements, Edc3p, and Mex67p
Source: PLoS Biol. 2010 Apr 27;8(4):e1000360. doi: 10.1371/journal.pbio.1000360 (PMC2864733; doi:10.1371/journal.pbio.1000360)
Supplement: Table S2 — Plasmids used in this study. (0.10 MB PDF) [file pbio.1000360.s010.pdf]

Name	Allele	Description
SYE74	pRS314-YRA1(XhoI-SacI)	Contains wild-type YRA1allele as a 2.2 kb XhoI-SacI fragment
SYE444	pRS314-YRA1-N-942(F16N)	Same as SYE74 but contains an internal deletion from nt 943 to the 3' end of the YRA1 intron
SYE495	pRS314-YRA1-N-852(NR9-F16N)	Same as SYE74 but contains an internal deletion from nt 853 to the 3' end of the YRA1 intron
SYE493	pRS314-YRA1-N-772(NR7-F16N)	Same as SYE74 but contains an internal deletion from nt 772 to the 3' end of the YRA1 intron
SYE521	pRS314-YRA1-N-753(R6A-F16N)	Same as SYE74 but contains an internal deletion from nt 754 to the 3' end of the YRA1 intron
SYR568	pRS314-YRA1-N-742(R6C-F16N)	Same as SYE74 but contains an internal deletion from nt 745 to the 3' end of the YRA1 intron
SYE491	pRS314-YRA1-N-712NR5-F16N)	Same as SYE74 but contains an internal deletion from nt 712 to the 3' end of the YRA1 intron
SYE487	pRS314-YRA1-N-542(NR1-F16N)	Same as SYE74 but contains an internal deletion from nt 544 to the 3' end of the YRA1intron
SYE401	pRS314-YRA1-N-400(F16)	Same as SYE74 but contains an internal deletion from nt 400 to the 3' end of the YRA1 intron
SYE436	pRS314-YRA1-N-372(F16-R3)	Same as SYE74 but contains an internal deletion from nt 373 to the 3' end of the YRA1 intron
SYE434	pRS314-YRA1-N-342(F16-R2)	Same as SYE74 but contains an internal deletion from nt 343 to the 3' end of the YRA1 intron
SYE403	pRS314-YRA1-N-311(F16-R1)	Same as SYE74 but contains an internal deletion from nt 313 to the 3' end of the YRA1 intron
SYE442	pRS314-YRA1-C-395(R4)	Same as SYE74 but contains an internal deletion from the 5' end of the YRA1 intron to nt 396
SYE497	pRS314-YRA1-C-543(R4-F1)	Same as SYE74 but contains an internal deletion from the 5' end of the YRA1 intron to nt 543
SYE648	pRS314-YRA1-C-548(R4-F1C)	Same as SYE74 but contains an internal deletion from the 5' end of the YRA1 intron to nt 548
SYE564	pRS314-YRA1-C-553(R4-F1A)	Same as SYE74 but contains an internal deletion from the 5' end of the YRA1 intron to nt 552
SYE650	pRS314-YRA1-C-559(R4-F1D)	Same as SYE74 but contains an internal deletion from the 5' end of the YRA1 intron to nt 558
SYE566	pRS314-YRA1-C-565(R4-F1B)	Same as SYE74 but contains an internal deletion from the 5' end of the YRA1 intron to nt 564
SYE611	pRS314-YRA1-C-625(R4-F14A)	Same as SYE74 but contains an internal deletion from the 5' end of the YRA1 intron to nt 624
SYE614	pRS314-YRA1-C-637(R4-F14B)	Same as SYE74 but contains an internal deletion from the 5' end of the YRA1 intron to nt 636
SYE503	pRS314-YRA1-C-648R4-F15)	Same as SYE74 but contains an internal deletion from the 5' end of the YRA1 intron to nt 648
SYE616	pRS314-YRA1-C-660(R4-F15A)	Same as SYE74 but contains an internal deletion from the 5' end of the YRA1 intron to nt 660
SYE618	pRS314-YRA1-C-672(R4-F15B)	Same as SYE74 but contains an internal deletion from the 5' end of the YRA1 intron to nt 672

SYE654	pRS314-YRA1-C-678(R4-F15C)	Same as SYE74 but contains an internal deletion from the 5' end of the YRA1 intron to nt 678
SYE477	pRS314-YRA1-C-683R4-F2)	Same as SYE74 but contains an internal deletion from the 5' end of the YRA1 intron to nt 684
SYE479	pRS314-YRA1-C-713(R4-F5)	Same as SYE74 but contains an internal deletion from the 5' end of the YRA1 intron to nt 714
SYE481	pRS314-YRA1-C-773(R4-F7)	Same as SYE74 but contains an internal deletion from the 5' end of the YRA1 intron to nt 774
SYE483	pRS314-YRA1-C-853(R4--F9)	Same as SYE74 but contains an internal deletion from the 5' end of the YRA1 intron to nt 852
SYE485	pRS314-YRA1-C-943(R4-F12)	Same as SYE74 but contains an internal deletion from the 5' end of the YRA1 intron to nt 942
SYE830	pRS314-YRA1-R-AC(AR3-F2-NR6C)	Same as SYE74 but contains internal deletions from nts 373-672 and nt 744 to the 3' end of the YRA1 intron
SYE558	pRS314-YRA1-R-BC(I-F1A-R6A)	Same as SYE74 but contains internal deletions from the 5' end to nt 552 and nt 754 to the 3' end of the YRA1 intron
SYE764	pRS314-YRA1-R-BCD(I-F1-F16N)	Same as SYE74 but contains internal deletions from the 5' end to nt 542 and nt 942 to the 3' end of the YRA1 intron
SYE646	pRS314-YRA1-R-CD(I-F15B-F16N)	Same as SYE74 but contains internal deletions from the 5' end to nt 672 and nt 942 to the 3' end of the YRA1 intron
SYE585	pRS314-YRA1-SL31	Same as SYE74 but contains a stem-loop structure 31 nucleotides downstream form the YRA1 transcription start site
SYE125	pRS314-YRA1-F7	Same as SYE74 but contains an internal deletions from nts 400-774
SYE591	pRS314-YRA1-SL31-N-400(F16)	Same as SYE401 but contains a stem-loop structure 31 nucleotides downstream form the YRA1 transcription start site
SYE603	pRS314-YRA1-SL31-C-773(R4-F7)	Same as SYE487 but contains a stem-loop structure 31 nucleotides downstream form the YRA1 transcription start site
SYE374	pRS314-YRA1-R1-F7	Same as SYE74 but contains internal deletions from nts 312-774
SYE710	pRS314-YRA1-F7-MS2	Same as SYE125 but contains two MS2 binding sites between the intron deletion sites
SYE389	pRS314-YRA1-R1-F7-MS2	Same as SYE374 but contains two MS2 binding sites between the intron deletion sites
SYE725	pRS314-YRA1-R1-F12-MS2	Same as SYE135 but contains two MS2 binding sites between the intron deletion sites
SYE712	pRS314-YRA1-N400-MS2	Same as SYE401 but contains two MS2 binding sites between the intron deletion sites
SYE393	pRS315-ADH1p-MS2p-Mex67p	Coding sequences of Mex67p fused to MS2-coat protein under the control of the ADH1 promoter
SYE454	pRS315-ADH1p-MS2p-Sub2p	Coding sequences of Sub2p fused to MS2-coat protein under the control of the ADH1 promoter
SYE686	pRS314-HA-MEX67	contains the HA-tagged MEX67 allele
SYE541	pRS314-YRA1-I-R4-NR6A	Same as SYE74 but contains an internal deletions from nts 286-394 and nt 754 to the 3' end of the YRA1 intron

SYE543	pRS314-YRA1-I-R4-NR6B	Same as SYE74 but contains an internal deletions from nts 286-394 and nt 734 to the 3' end of the YRA1 intron
SYE539	pRS314-YRA1-I-R4-NR5	Same as SYE74 but contains an internal deletions from nts 286-394 and nt 714 to the 3' end of the YRA1 intron
SYE537	pRS314-YRA1-I-R4-NR2	Same as SYE74 but contains an internal deletions from nts 286-394 and nt 684 to the 3' end of the YRA1 intron
SYE549	pRS314-YRA1-I-F1-R6A	Same as SYE74 but contains an internal deletions from nts 286-542 and nt 754 to the 3' end of the YRA1 intron
SYE560	pRS314-YRA1-I-F1B-R6A	Same as SYE74 but contains an internal deletions from nts 286-564 and nt 754 to the 3' end of the YRA1 intron
SYE547	pRS314-YRA1-I-F13-R6A	Same as SYE74 but contains an internal deletions from nts 286-577 and nt 754 to the 3' end of the YRA1 intron
SYE107	pRS314-YRA1-R1	Same as SYE74 but contains internal deletions from nts 312-394
SYE171	pRS314-YRA1-R2	Same as SYE74 but contains internal deletions from nts 342-394
SYE173	pRS314-YRA1-R3	Same as SYE74 but contains internal deletions from nts 372-394
SYE99	pRS314-YRA1-F1	Same as SYE74 but contains internal deletions from nts 400-542
SYE175	pRS314-YRA1-F13	Same as SYE74 but contains internal deletions from nts 400-577
SYE177	pRS314-YRA1-F14	Same as SYE74 but contains internal deletions from nts 400-612
SYE179	pRS314-YRA1-F15	Same as SYE74 but contains internal deletions from nts 400-647
SYE121	pRS314-YRA1-F5	Same as SYE74 but contains internal deletions from nts 400-712
SYE123	pRS314-YRA1-F6	Same as SYE74 but contains internal deletions from nts 400-743
SYE127	pRS314-YRA1-F8	Same as SYE74 but contains internal deletions from nts 400-797
SYE135	pRS314-YRA1-F12	Same as SYE74 but contains internal deletions from nts 400-943
SYE1103	pRS314-HA-YRA1-C672(R4-15B)	Same as SYE 618 but contains HA tag at the beginning of YRA1 exon1
SYE1105	pRS314-HA-YRA1-C773(R4-F7)	Same as SYE 481 but contains HA tag at the beginning of YRA1 exon1
SYE1107	pNOPGFP-MTR2	From Dr. Ed Hurt
SYE1108	pRS315-mtr2-9	From Dr. Ed Hurt
SYE1109	pRS315-mtr2-21	From Dr. Ed Hurt
SYE1110	pRS315-mtr2-26	From Dr. Ed Hurt
