# Supplementary material for: Degradation of YRA1 Pre-mRNA in the Cytoplasm Requires Translational Repression, Multiple Modular Intronic Elements, Edc3p, and Mex67p
Source: PLoS Biol. 2010 Apr 27;8(4):e1000360. doi: 10.1371/journal.pbio.1000360 (PMC2864733; doi:10.1371/journal.pbio.1000360)
Supplement: Table S3 — Oligonucleotides used in this study. (0.09 MB PDF) [file pbio.1000360.s011.pdf]

Name	Sequences
YRA1-3	ACTTGAAGAACTATAAAAGGCCGC
YRA1-4	GTGTGCCATATCCTTCCTTACAAA
YRA1-5	GTATGTTAATACGTGAAATGAGAGCT
YRA1-6	GAGTTGCCAAGCTCTTGGACACCACTA
YRA1-7	TCATGAAAGACATGTTTCCCATAGCTA
YRA1-p1	AGCTCTCATTTCACGTATTAACATAC
YRA1-BglII-F	ACCTAGATCTGCTAAATGTCTGCTAACTTAGATA
YRA1-BglI-R	TAGCAGATCTAGGTATTTTCTTAATATGGATTTCTTA
YRA1-StyI-R	TGCTTAATGTCCCTTGGCAAACCTTCG
YRA1-DU-R2	TAGCAGATCTTTATCTATTTGTCTTCGATAGTTTTCG
YRA1-DI-5’	GATCGAATTCCTCCTTGATTGTTTGTGTATTGTCC
YRA1-DI-F1	GATCGAATTCAGAATGGGTTTGTTCTTATTGGCTG
YRA1-DI-F1A	GATCGAATTCTGTTCTTATTGGCTGGGTTAACTCAAAT
YRA1-DI-F1B	GATCGAATTCCTGGGTTAACTCAAATCAATCTGACTAT
YRA1-DI-F1C	GATCGAATTCGGGTTTGTTCTTATTGGCTGGGTTAAC
YRA1-DI-F1D	GATCGAATTCTATTGGCTGGGTTAACTCAAATCAATC
YRA1-DI-F2	GATCGAATTCCCAATACTGAAGTTGTGGAAGGATC
YRA1-DI-F5	GATCGAATTCATTTTGTATCCTTTCAAGAGGGAGT
YRA1-DI-F6	GATCGAATTCTCAGTTTCGAATGGCACCATTTTCA
YRA1-DI-F7	GATCGAATTCTAGCTATGGGAAACATGTCTTTCAT
YRA1-DI-F9	GATCGAATTCAAAATATTTGATTTGAGACACCTAA
YRA1-DI-F12	GATCGAATTCATGGTATACTTTTAATACTCTCAA
YRA1-DI-F13	GATCGAATTCAATCAATCTGACTATCTGCTAATCT
YRA1-DI-F14	GATCGAATTCCATTTTTAAGCTGGCGTATTGTGTC
YRA1-DI-F14A	GATCGAATTCGGCGTATTGTGTCGCCTCGACCGTGATA
YRA1-DI-F14B	GATCGAATTCCGCCTCGACCGTGATAGTTAGTTCCTAT
YRA1-DI-F15	GATCGAATTCTGATAGTTAGTTCCTATTTTTAATA
YRA1-DI-F15A	GATCGAATTCCCTATTTTTAATAAGCGTATCATCCAAT
YRA1-DI-F15B	GATCGAATTCAAGCGTATCATCCAATACTGAAGTTGTG
YRA1-DI-F15C	GATCGAATTCATCATCCAATACTGAAGTTGTGGAAGG
YRA1-DI-F16N	TACGCCATGGGAATTTTTTGCATCTCAAGTAGGTGGTG
YRA1-DI-F16	GATCGAATTCGAATTTTTTGCATCTCAAGTAGGTGGTG
YRA1-DI-3’	AATCTTGATCTGCCTCCATCGATTG
YRA1-DI-R1	GATCGAATTCAGCTCTCATTTCACGTATTAACATA
YRA1-DI-R2	GATCGAATTCTTAGTTACTCGCATCGTCTCGTGTG
YRA1-DI-R3	GATCGAATTCGAGAAATATTCTTTGTAAGGAAGGA
YRA1-DI-R4	GATCGAATTCTCTTACAGCATCCTGCTTAATGTCC
YRA1-R4-BamHI	TACGGGATCCTCTTACAGCATCCTGCTTAATGTCC
YRA1-EcoR1-F	GATCAAGAATTCTCTTTTTAGAGATGAA
YRA1-NcoI-F	TACGCCATGGTATACTTTTAATACTCTCAACTC
YRA1-NcoI-R	TATACCATGGCGTAGGAAAAATAAACCCTCAAATAA
YRA1-NcoI-R9	TACGCCATGGTGTAACTATCATAATAAAAAAATGAAG
YRA1-NcoI-R7	TACGCCATGGTTTTTTGAAAATGGTGCCATTCGAAAC
YRA1-NcoI-R5	TACGCCATGGTAATTGATCCTTCCACAACTTCAGTAT
YRA1-NcoI-R2	TACGCCATGGATGATACGCTTATTAAAAATAGGAACT
YRA1-NcoI-R1	TACGCCATGGCAGTGGTAGCTGGTAATTCCAACCTTA
YRA1-NcoI-R6A	TACGCCATGGTCGAAACTGAAAGCTACTCCCTCTTGA
YRA1-NcoI-R6B	TACGCCATGGCTCTTGAAAGGATACAAAATTAATTGA

YRA1-NcoI-R6C	TACGCCATGGAAGCTACTCCCTCTTGAAAGGATACAAA
YRA1-SL31-F	GATCGGATCCCGCGGTTCGCCGCGGGATCTAAAAATCTCCCTCGTTCTATTTGAAACTTT
YRA1-SL31P	GCGGCGAACCGCGGGATCTTTATCTAT
YRA1-MS2-I-R	GATCGAATTCGATGGATCCAGCTCTCATTTCACGTATTAACATACTCTT
MS2-BamH-EcoR-F	GATCCAAAGTTGAGGATCACCCAACTGTGCAGGTCGAGACTAGAAAAGTTGAGGATCACCCAACTG
MS2-BamH-EcoR-R	AATTCAGTTGGGTGATCCTCAACTTTTCTAGTCTCGACCTGCACAGTTGGGTGATCCTCAACTTTG
YRA1-BamHI-R	GATCGGATCCTTGATCAAAAGCGTGTGCCATATCCTTC
YRA1-RTF1	AGCCTTCCAAACAGAAGAGGCCCTATC
YRA1-RTR1	AAGTATTTCGGACTCTTTCTTCATCTC
CYH2-RTF1	AAGACTAGAAAGCACAGAGGTCACGTC
CYH2-RTR1	AGTATGACAAAGGCTATGCACACTTTC
MS2-MEX67-F	GATCGCGGCCGCGCTAGCATGAGCGGATTTCACAATGTTGGAAATA
MS2-MEX67-R	GATCGTCGACGAGCTCATCAAAAGCTCTCTTAGATCTTTGAA
MEX67-5UTR-F	GATCGCGGCCGCAGCGATGCAGATGTGTATAAAACTGTCA
MEX67-5UTR-R	GATCCCATGGTTTTTTTGCTGCTATTAGCGATGCAGAATT
MEX67-F-S1	CAGAGATTGTCAATCGGGCAAGAGTCC
MEX67-F-S2	GACAACCAACTTAAGGACATCTCAGCA
SUB2-MS2-F	GATCGCGGCCGCGCTAGCATGTCACACGAAGGTGAAGAAGATTTAT
SUB2-MS2-R	GATCGTCGACTCTAGATTCCAAAAATCTTACCGTTAAGAA
YRA1-NotI	GATCGCGGCCGCCTCGAGGGTATATTAAAGCTATTTTACCACT
YRA1-HA-NcoI-R	GATCCCATGGTTAGCAGATGTAGGTATTTTCTTAATAT
YRA1-Nhe-F	CGGTGCTAGCATGGCTAACTTAGATAAATCCTTAGACGAAATC
